# Alterations of proteome, mitochondrial dynamic and autophagy in the hypothalamus during activity-based anorexia

**DOI:** 10.1038/s41598-018-25548-9

**Published:** 2018-05-08

**Authors:** Séverine Nobis, Alexis Goichon, Najate Achamrah, Charlène Guérin, Saida Azhar, Philippe Chan, Aline Morin, Christine Bôle-Feysot, Jean Claude do Rego, David Vaudry, Pierre Déchelotte, Liliana Belmonte, Moïse Coëffier

**Affiliations:** 1 Normandie University, UNIROUEN, INSERM Unit 1073, Rouen, France; 2 Normandie University, UNIROUEN, Institute for Research and Innovation in Biomedicine (IRIB), Rouen, France; 3 Rouen University Hospital, Nutrition Department, Rouen, France; 4 Normandie University, UNIROUEN, Platform in proteomics PISSARO, Rouen, France; 5 Normandie University, UNIROUEN, Animal Behaviour Platform SCAC, Rouen, France; 6 Normandie University, UNIROUEN, INSERM Unit 1239, Mont-Saint-Aignan, France

## Abstract

Restrictive anorexia nervosa is associated with reduced eating and severe body weight loss leading to a cachectic state. Hypothalamus plays a major role in the regulation of food intake and energy homeostasis. In the present study, alterations of hypothalamic proteome and particularly of proteins involved in energy and mitochondrial metabolism have been observed in female activity-based anorexia (ABA) mice that exhibited a reduced food intake and a severe weight loss. In the hypothalamus, mitochondrial dynamic was also modified during ABA with an increase of fission without modification of fusion. In addition, increased dynamin-1, and LC3II/LC3I ratio signed an activation of autophagy while protein synthesis was increased. In conclusion, proteomic analysis revealed an adaptive hypothalamic protein response in ABA female mice with both altered mitochondrial response and activated autophagy.

## Introduction

Anorexia Cachexia Syndrome (ACS) is characterized by a reduced food intake and a body weight loss due to a reduction of fat and lean mass^1^. ACS is mainly observed in patients with chronic inflammatory diseases and cancer^2^ but has also been described in severe patients with Anorexia Nervosa (AN)^3^. In patients with AN, a low grade inflammatory response has been reported^4^. AN is a severe eating disorder defined by low body weight associated with an intense fear of gaining weight and misshapen cognitions concerning weight, shape, and drive for thinness^5^. AN is a major public health problem with an increased incidence, a female predominance and a lifetime prevalence estimated between 0.3% and 0.7%^6–8^ that is constantly increasing in different countries^9^. AN is a multifactorial disease^10^ characterized by the highest mortality rate of all psychiatric disorders^11^ and a high relapse rate^12^. Anorexia contributes to the appearance of protein-energy malnutrition and low-grade inflammatory states^13^ leading to cachexia.

Hypothalamus is a key centre in the regulation of food intake and energy homeostasis^14,15^. In response to peripheral and hormonal signals, hypothalamic neuronal activity and transmitter release are modified such as orexigenic and anorexigenic neuropeptide release leading to the regulation of food intake and energy homeostasis^16^. Recent papers also underlined the role of mitochondrial dynamic and autophagy in the regulation of hypothalamic neuron activity and feeding behaviour^17–20^. Proteomic profiling of the hypothalamus have been performed in different animal models to study body weight homeostasis. Indeed, hypothalamic proteome alterations have been described in cancer-induced anorexia-cachexia^21^ or in LPS-induced anorexia^22^, while other authors underlined the role of hypothalamic leptin signaling^23^. Nevertheless, to our knowledge, there was no study evaluating hypothalamic proteome in the commonly used animal model, the activity-based anorexia (ABA) model. Interestingly, in AN patients, an excessive physical activity is frequently observed^24^ that enhances the relevance of the ABA model^25^. ABA paradigm was initially developed with food access limited to 1 h or 2 h per day^26^ and then optimized in mice with a progressive decrease of time access to food^27^. We have chosen this latter paradigm and we reproduced it in male and female mice^28–30^.

Thus, in the present paper, we aimed to evaluate hypothalamic proteome and then identified altered biological process in ABA model associating a spontaneous reduced food intake and a severe body weight loss.

## Results

Mice were randomized into 3 groups: an activity-based anorexia group (ABA), a limited-food access group (LFA) and an ad libitum group (Control). ABA procedure was performed as previously described^28–30^. Briefly, ABA mice were placed in cages with an activity wheel, while both LFA and Control mice were placed in standard cages. From day 6, LFA and ABA mice had a progressive limited access to food.

### Physical activity, body weight, body composition and food intake

**Figure 1:**
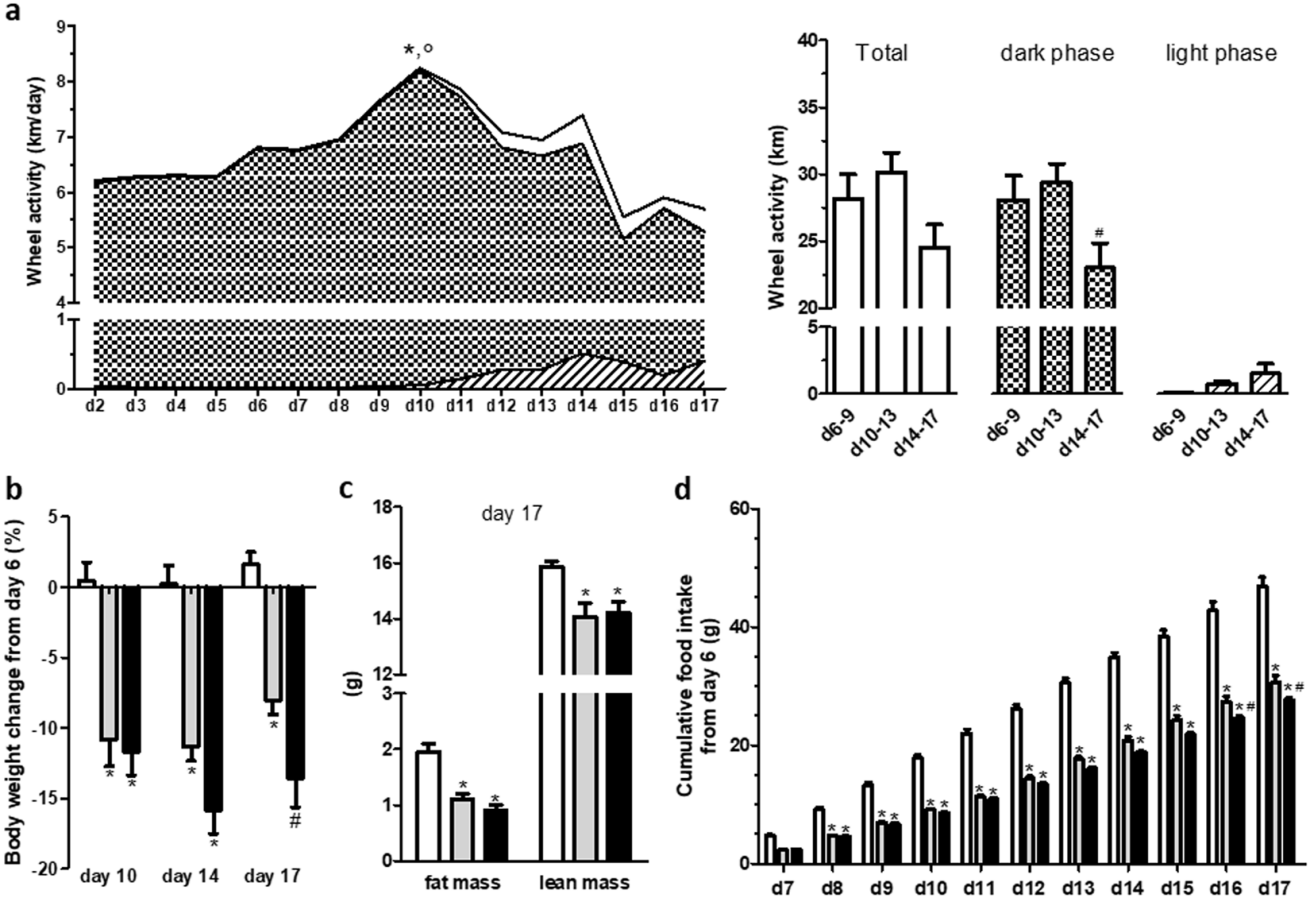
Wheel activity, body weight, body composition and cumulative food intake. (**a**) Wheel activity, expressed in km/d or km, in activity-based anorexia mice, was monitored by using RunningWheel® software (Intellibio). Total activity (white area and bars) and activities in the dark (dotted area and bars) or light (hatched area and bars) phase were continuously measured. *p < 0.05 *vs* d5 and °p < 0.05 vs d17 for total activity. ^#^p < 0.05 vs d10-13 for dark phase activity (n = 8/group). (**b**) Body weight change at d10, d14 and d17, (**c**) body composition measured at d17 and (**d**) cumulative food intake after the beginning of limitation of food access measured in Control mice (open bars) or in mice with limitation of food access (LFA, grey bars) or activity-based anorexia (ABA, black bars). (**b**–**d**) *p < 0.05 *vs* Control. ^#^p < 0.05 *vs* LFA (n = 8/group).

After the beginning of progressive limited food access, physical activity increased until day 10 that was mainly related to physical activity performed during dark phase (Fig. 1a). Then, both total and dark wheel activities decreased while physical activity during light phase appeared and increased until day 17, even if light wheel activity level remained low (Fig. 1a). However, total wheel activity remained higher than 5 km/day at d17 that is a higher value than previously observed in male mice^28^.

Body weight loss was similar between LFA and ABA mice at d10 (p < 0.05 vs Control). At d14 and d17, body weight loss was more marked in ABA mice compared with LFA and difference reached significance at d17 (p < 0.05; Fig. 1b). At d17, fat and lean mass were similarly reduced in ABA and LFA groups compared with Control group (Fig. 1c). In addition, LFA and ABA mice exhibited reduced protein synthesis and increased LC3II/LC3I ratio, a marker of autophagy, in soleus (both p < 0.05) but not in anterior tibialis (Supplemental Fig. S1).

Both ABA and LFA mice exhibited lower daily food intake than Control mice (data not shown). Cumulative food intake was significantly decreased in ABA and LFA female mice compared with Control mice from d8 until the end of experiment (Fig. 1d). Furthermore, at d16 and d17, ABA mice exhibited a lower cumulative food intake compared with LFA mice (p < 0.05). Water intake in ABA and LFA groups was not affected by limited food access (data not shown). Interestingly, in an additional experiment, we observed that food intake during the first hour at day 17 was significantly lower in ABA mice than in LFA mice when all mice were placed in standard cages without activity wheel (Supplemental Fig. S2).

As we previously reported that female ABA mice exhibited inflammatory response^30^, we considered that ABA procedure led to cachectic mice exhibiting reduced spontaneous food intake. We thus analyzed the proteome of hypothalamus, which is a major centre in the regulation of food intake and energy homeostasis.

### Hypothalamic proteome during initial (d10) and persistent weight loss (d17)

**Figure 2:**
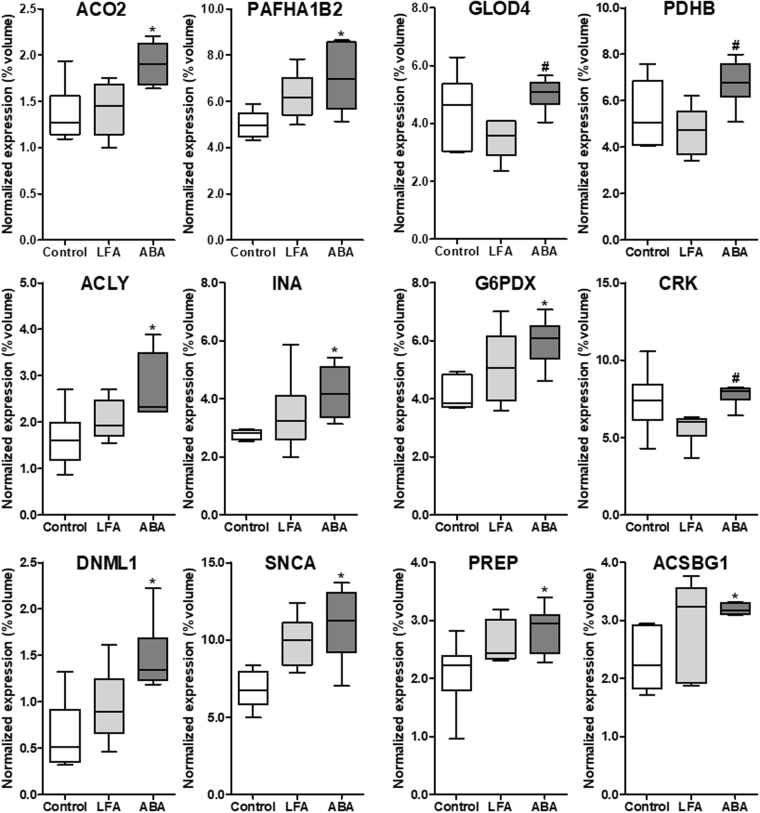
Hypothalamic proteins differentially expressed at day 10. Twelve differently modulated proteins were identified at d10 (n = 6/group). *p < 0.05 *vs* Control. ^#^p < 0.05 vs LFA. ACO2: Aconitate hydratase, mitochondrial precursor; PAFAH1B2: Platelet-activating factor acetylhydrolase IB subunit beta; GLOD4: Glyoxalase domain-containing protein 4; PDHB: Pyruvate dehydrogenase E1 component subunit beta, mitochondrial; ACLY: ATP-citrate synthase; INA: Alpha-internexin; G6PDX: Glucose-6-phosphate 1-dehydrogenase X; CRK: Adapter molecule Crk; DNM1L: Dynamin-1-like protein; SNCA: Alpha-synuclein; PREP: Prolyl endopeptidase; ACSBG1: Long chain fatty acid coA ligase.

**Figure 3:**
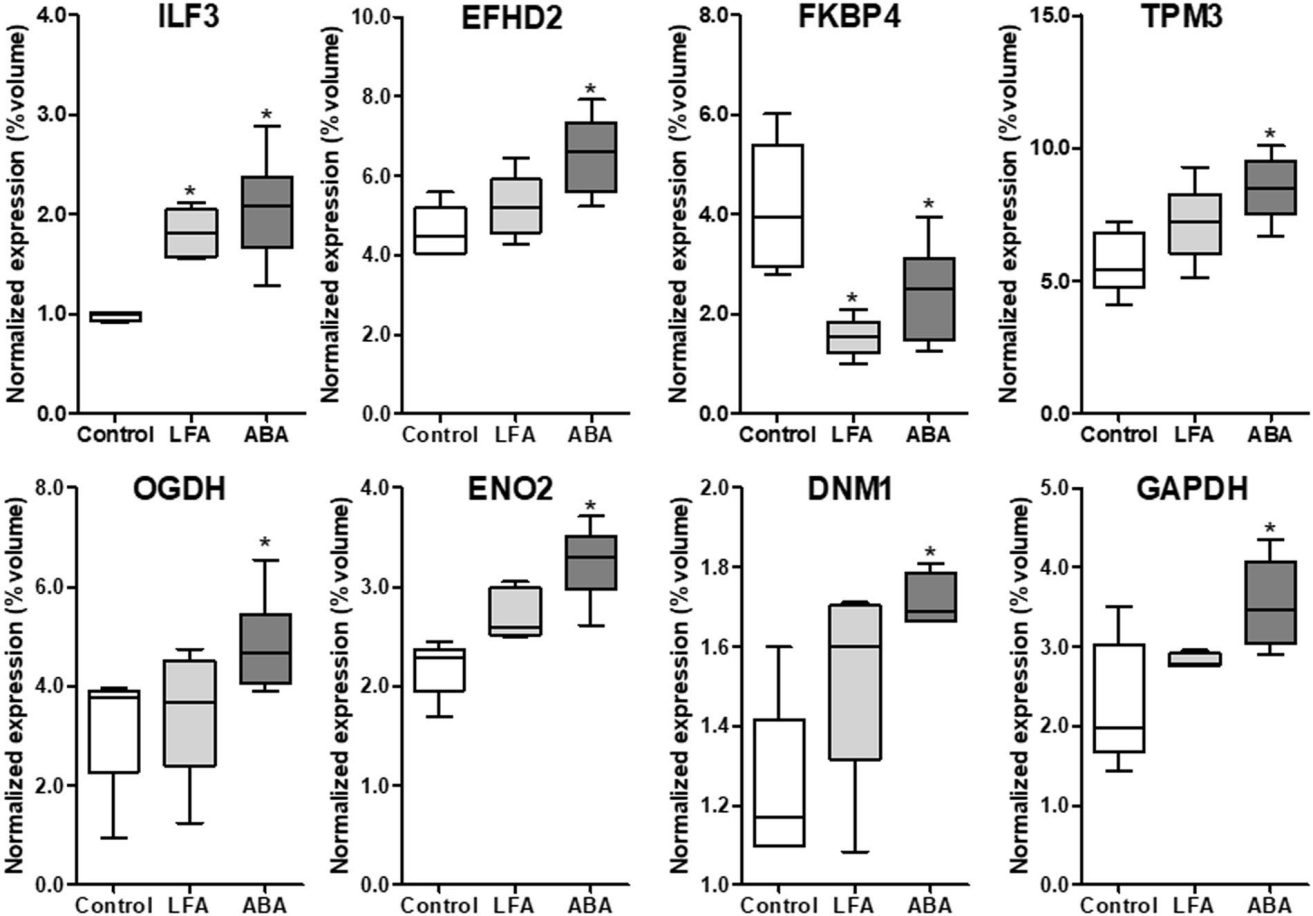
Hypothalamic proteins differentially expressed at day 17. Eight differently modulated proteins were identified at d17 (n = 6/group). *p < 0.05 *vs* Control. ILF3: Interleukin enhancer-binding factor 3; EFHD2: EF-hand domain-containing protein D2; FKBP4: Peptidyl-prolyl cis-trans isomerase; TPM3: Tropomyosin alpha-3 chain; OGDH: 2-Oxoglutarate dehydrogenase, mitochondrial; ENO2: Gamma-enolase; DNM1: Dynamin-1; GAPDH: Glyceraldehyde-3-phosphate dehydrogenase.

**Figure 4:**
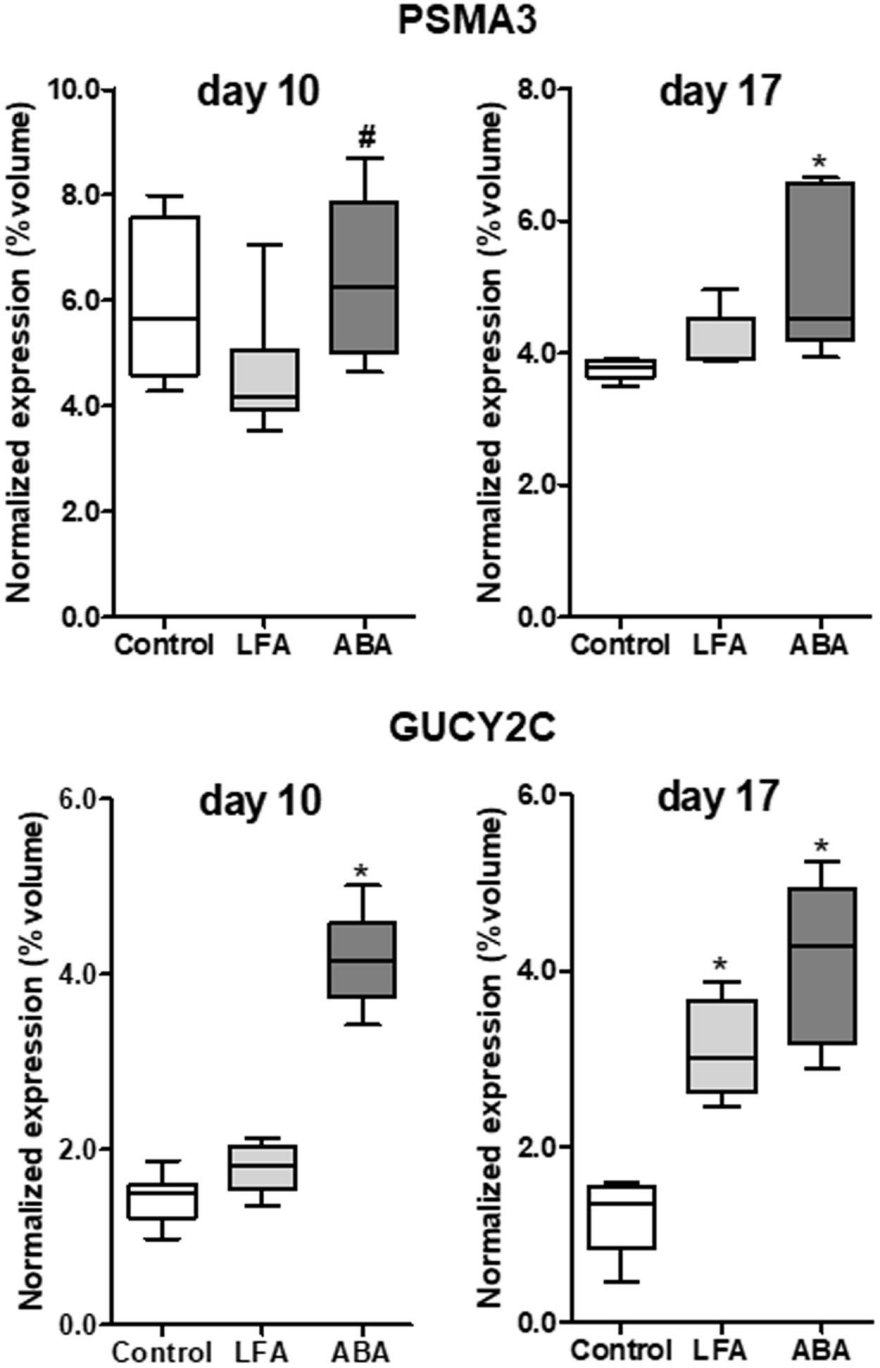
Hypothalamic proteins differentially expressed both at day 10 and day 17. Two differently modulated proteins were identified both at d10 and d17 (n = 6/group at each time). *p < 0.05 *vs* Control. ^#^p < 0.05 vs LFA. PSMA3: Proteasome subunit alpha type 3; GUCY2C: Heat stable enterotoxin receptor.

At d10 and d17, thirty-four protein spots were differentially and significantly expressed between the three groups (p < 0.05; at least ± 1.4 fold modulated). After mass spectrometry, eleven spots remained undetermined and two protein spots corresponded to same protein identification. Finally, twenty-two non-redundant proteins were identified (Supplemental Fig. S3). Among these proteins, twelve, eight and two proteins were, respectively, modified at d10, at d17 and both at d10 and d17 (Table 1; Figs 2–4).

**Table 1:** Identified proteins altered at d10 and/or d17 in the hypothalamus.

Spots’ labels	SwissProt accession number	Protein name	Short name	pI	MW (Da)	Score on MASCOT^a^	Sequence coverage	Peptide hit^b^
A^c^	Q99KI0	Aconitate hydratase, mitochondrial	ACO2	8.08	85,464	827	31%	7
B	Q61206	Platelet-activating factor acetylhydrolase IB subunit beta	PAFAH1B2	5.57	25,581	148	10%	5
C	Q9CPV4	Glyoxalase domain-containing protein 4	GLOD4	5.28	33,317	247	27%	6
D	Q9D051	Pyruvate dehydrogenase E1 component subunit beta, mitochondrial	PDHB	6.41	38,937	300	29%	5
E	Q91V92	ATP-citrate synthase	ACLY	7.13	119,728	450	19%	7
F	P46660	Alpha-internexin	INA	5.35	55,383	1719	49%	16
G	Q00612	Glucose – 6 – phosphate 1 – dehydrogenase X	G6PDX	6.06	59,263	477	21%	5
H	Q64010	Adapter molecule Crk	CRK	5.38	33,815	186	19%	4
I	Q8K1M6	Dynamin − 1 – like protein	DNM1L	6.61	82,658	652	29%	11
J	O55042	Alpha – synuclein	SNCA	4.74	14,485	245	49%	4
K	Q9QUR6	Prolyl endopeptidase	PREP	5.44	80,700	1354	50%	16
L	Q99PU5	Long chain fatty acid coA ligase	ACSBG1	5.67	80, 374	838	27%	15
M	Q9Z1X4	Interleukin enhancer-binding factor 3	ILF3	8.86	96,021	304	12%	6
N	Q9D8Y0	EF-hand domain-containing protein D2	EFHD2	5.01	26,791	332	32%	4
O	P30416	Peptidyl-prolyl cis-trans isomerase	FKBP4	5.54	51,572	275	27%	8
P	P21107	Tropomyosin alpha-3 chain	TPM3	4.68	29,021	358	31%	10
Q	Q60597	2 - Oxoglutarate dehydrogenase, mitochondrial	OGDH	6.36	116,449	656	31%	18
R	P17183	Gamma – enolase	ENO2	4.99	47,297	891	56%	14
S	P39053	Dynamin – 1	DNM1	7.61	97,803	234	19%	3
T	P16858	Glyceraldehyde - 3 – phosphate dehydrogenase	GAPDH	8.44	35,810	115	28%	8
U	O70435	Proteasome subunit alpha type 3	PSMA3	5.29	28,405	95	12%	3
V	Q3UWA6	Heat stable enterotoxin receptor	GUCY2C	6.38	123,232			

Proteins identified at d10 (A-L), at d17 (M-T) and both d10/d17 (U and V). ^a^MASCOT, algorithm that uses mass spectrometric data to identify proteins. ^b^Peptide hit corresponding to unique peptide. ^c^Protein spots corresponding to one protein identification. pI: Isoelectric point; MW: Molecular weight.

At d10, nine proteins were significantly increased in ABA mice compared with Controls: aconitate hydratase, mitochondrial (ACO2, Spot A), platelet-activating factor acetylhydrolase IB subunit beta (PAFAH1B2, Spot B), ATP-citrate synthase (ACLY, Spot E), alpha-internexin (INA, Spot F), glucose-6-phosphate 1 dehydrogenase X (G6PDX, Spot G), dynamin-1 like protein (DNM1L, Spot I), alpha-synuclein (SNCA, Spot J), prolyl endopeptidase (PREP, Spot K) and long chain fatty acid coA ligase (ACSBG1, Spot L). In addition, three proteins were significantly upregulated in ABA compared with LFA: glyoxalase domain-containing protein 4 (GLOD4, Spot C), pyruvate dehydrogenase E1 component subunit beta, mitochondrial (PDHB, Spot D) and adapter molecule Crk (CRK, Spot H, Table 1 and Fig. 2).

At d17, six proteins were significantly upregulated in ABA mice compared with Controls: EF-hand domain-containing protein D2 (EFHD2, Spot N), tropomyosin alpha-3 chain (TPM3, Spot P), 2-oxoglutarate dehydrogenase, mitochondrial (OGDH, Spot Q), gamma-enolase (ENO2, Spot R), dynamin-1 (DNM1, Spot S) and glyceraldehyde-3 phosphate dehydrogenase (GAPDH, Spot T). In addition, two proteins were significantly affected both in LFA and ABA groups compared with Control group: interleukin enhancer-binding factor 3 (ILF3, Spot M) that was upregulated and peptidyl-prolyl cis-trans isomerase (FKBP4, Spot O) that was downregulated (Table 1 and Fig. 3).

Finally, proteasome subunit alpha type 3 (PSMA3, Spot U) and heat stable enterotoxin receptor (GUCY2C, Spot V) were affected both at d10 and at d17 (Table 1 and Fig. 4). Interestingly, GUCY2C was significantly upregulated in ABA mice at d10 and d17 but only at d17 in LFA mice compared with Controls (Fig. 4).

**Figure 5:**
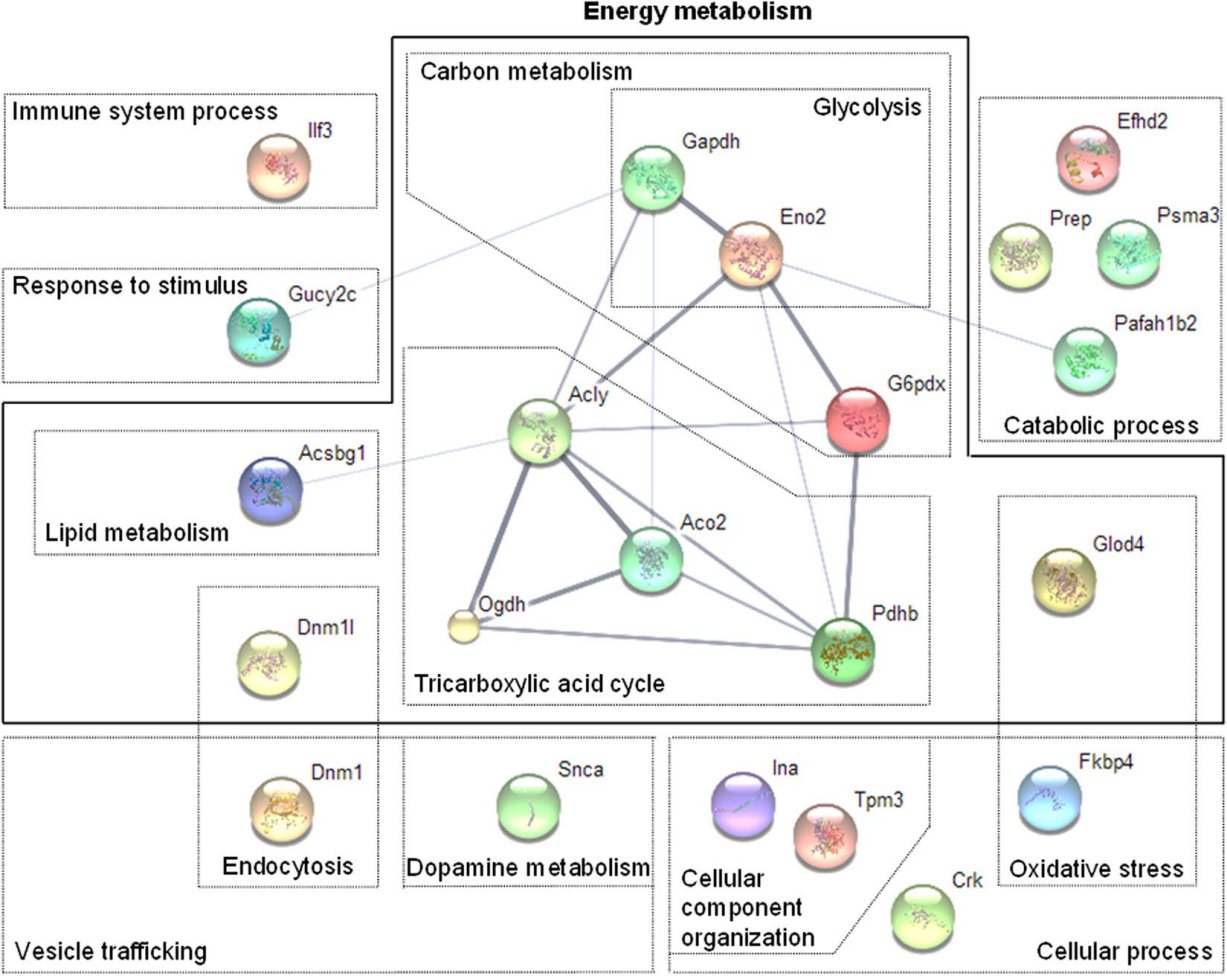
Non-exhaustive representation of proteins clustering analysis. The 22 proteins were analyzed using open source databases UniProt, PANTHER, KEGG and network program STRING, resulting in protein clusters. Proteins were classified according to their biological process including fourteen clusters (cellular component organization, cellular process, oxidative stress, dopamine metabolism, endocytosis, vesicle trafficking, lipid metabolism, tricarboxylic acid cycle, catabolic process, glycolysis, carbon metabolism, response to stimulus, immune system process and energy metabolism).

**Figure 6:**
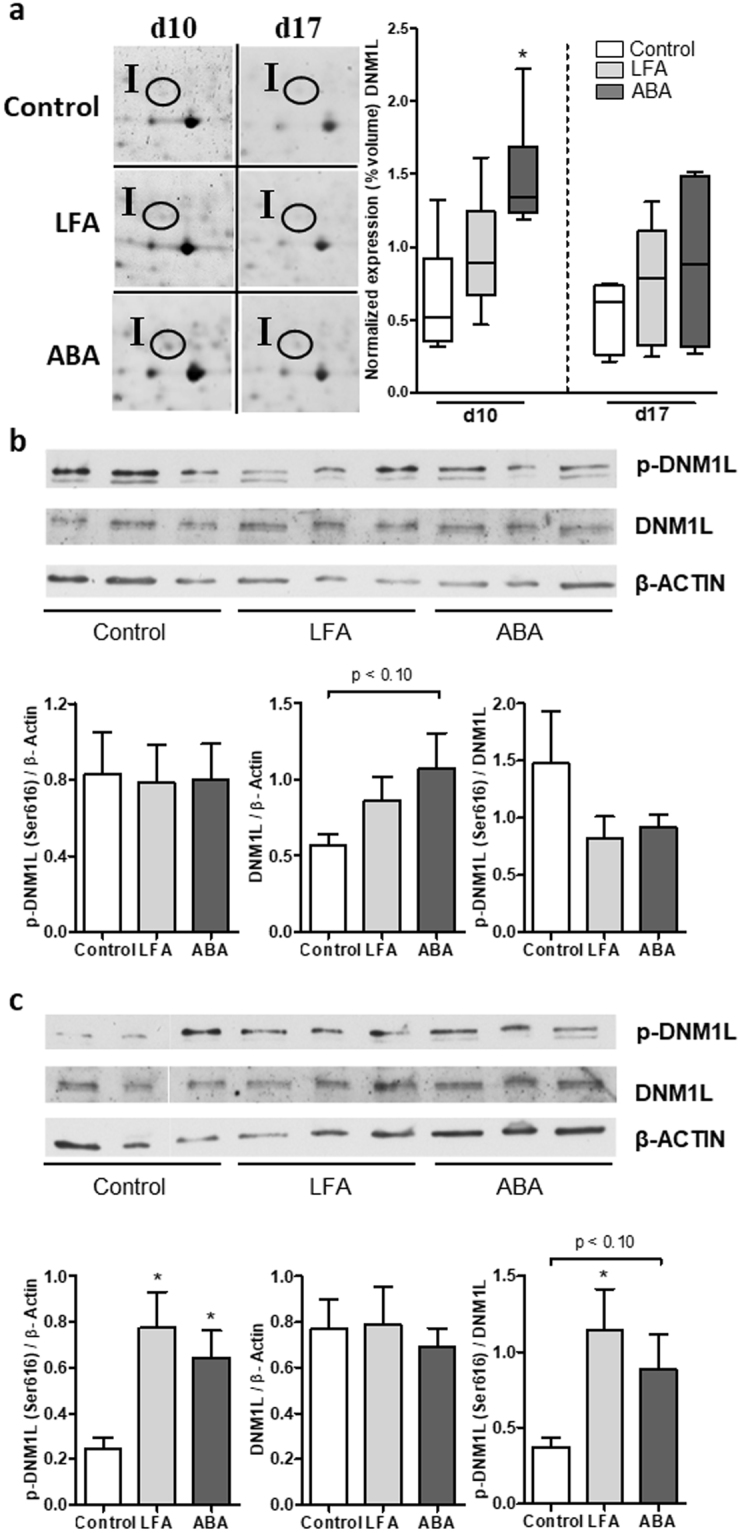
DNM1L hypothalamic expression. (**a**) Representative enlargements of silver-stained 2-dimensional gels (from n = 1/group) and proteomic expression profile for DNM1L protein at d10 and d17 in Control, LFA and ABA mice (n = 6/group at each time). *p < 0.05 *vs* Control. (**b**,**c**) Representative immunoblots (from 3 individuals/group) and densitometric analysis of phosphorylated-DNM1L at Ser^616^, total DNM1L, and β-actin at d10 (**b**) and d17 (**c**) (n = 6/group at each time).

All these proteins are involved in various biological pathways. By using STRING, UniProt- GO Biological Process, KEGG and PANTHER databases, functional clustering (Fig. 5) has been performed and revealed several clusters including protein involved in cellular process, cellular component organization, immune system process, response to stimulus, dopamine metabolism, energy metabolism, glycolysis, tricarboxylic acid cycle, lipid and carbon metabolism, oxidative stress, catabolic process, vesicle trafficking or endocytosis. Interestingly, among these altered proteins, 10 are involved in energy metabolism and were upregulated in ABA mice at d10 and/or d17 (p < 0.05, Fig. 5). For instance, G6PDX involved in the pentose phosphate pathway and ACLY, ACO2, PDHB involved in the tricarboxylic acid (TCA) cycle were upregulated at d10 (Fig. 2). GAPDH and ENO2 involved in glycolysis and OGDH involved in TCA cycle were increased in ABA mice at d17 (Fig. 3). These data suggest that energy metabolism seems to be increased in the hypothalamus of ABA mice. We thus analysed AMP-activated protein kinase (AMPK), a sensor of energy status, and one of its downstream targets, acetyl-CoA carboxylase (ACC). AMPK expression was not affected but the ratio p-AMPK/AMPK was significantly reduced in the hypothalamus of LFA and ABA mice at d17 (Supplemental Fig. S4). By contrast, ACC expression was increased in LFA mice compared with Controls but not in ABA mice while p-ACC/ACC ratio was only significantly increased in ABA mice (Supplemental Fig. S4). By proteomics, we also observed that DNM1L was increased in ABA mice compared with Controls (p < 0.05 at d10, p < 0.10 at d17, Fig. 6a), DNM1L regulating the mitochondria number by acting on fission mechanisms.

### Mitochondrial dynamics in the hypothalamus of ABA mice

As DNM1L activation is dependent of its phosphorylation on serine 616 residue (p-DNM1L(Ser616)), we assessed p-DNM1L and DNM1L expression by western blot analysis (Fig. 6b,c). At d10, we observed a trend for an increase of DNM1L expression in ABA mice compared with Controls, whereas p-DNM1L expression was not affected (Fig. 6b). Consequently, p-DNM1L/DNM1L ratio was reduced in ABA mice compared with Controls, even if difference did not reach significance. By contrast, at d17 DNM1L expression was not modified while p-DNM1L expression was significantly increased in ABA mice compared with Control mice (p = 0.03; Fig. 6c). Thus, p-DNM1L/DNM1L increased at d17 in ABA mice. By contrast, markers of mitochondrial fusion, mitofusin 1, mitofusin 2 and optic atrophy 1 (OPA 1), were not affected in LFA and ABA groups (Supplemental Fig. S5). In addition, mitochondrial biogenesis seemed to be unaffected at day 17 since PGC-1α and TFAM expression remained unchanged (Supplemental Fig. S6). All these data suggest an increase of mitochondrial fission in ABA mice at d17 while mitochondrial fusion and biogenesis remained unaffected.

To further investigate mitochondria, we assessed in the hypothalamus the Citrate Synthase (CS) Activity, a marker of the mitochondrial membrane surface. We did not observe significant difference between Control, LFA and ABA groups (Table 2) that was illustrated by the immunostaining of VDAC1, expressed at the outlier membrane of mitochondria (Supplemental Fig. S7). As previous data reported a role for both DNM1L and mitochondria fission in the regulation of autophagy^31^, we thus analysed autophagy in hypothalamus of ABA mice.

**Table 2:** Citrate synthase activity in hypothalamus of Control, LFA and ABA mice at d17.

	d17		
	Control	LFA	ABA
Citrate Synthase Activity (µmol/min/µg protein)	0.804 ± 0.060	0.732 ± 0.050	0.850 ± 0.076

Results are means ± SEM (from n = 8/group) and expressed in µmol/min/µg protein.

### Hypothalamic autophagy in ABA model at d17

**Figure 7:**
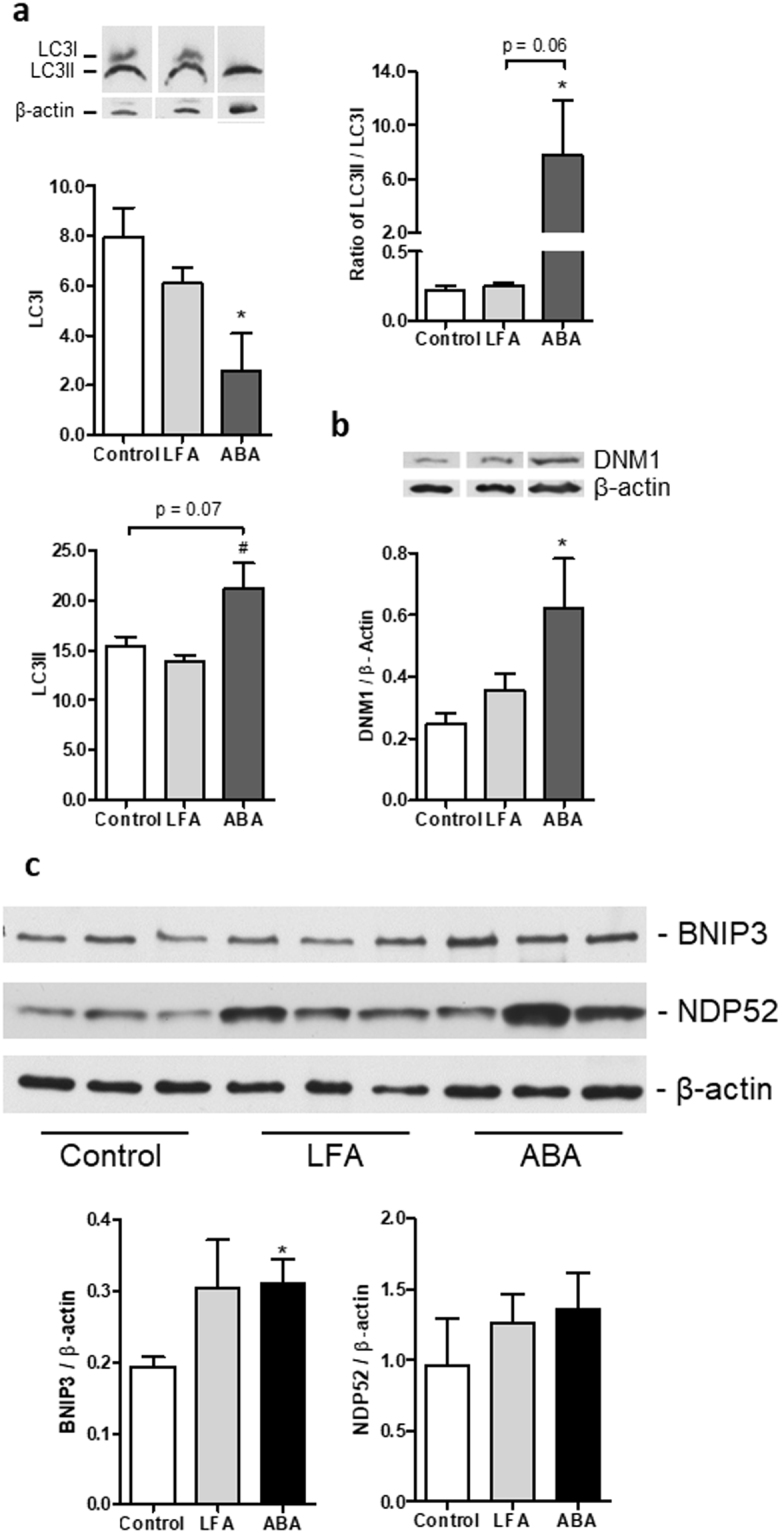
Hypothalamic autophagy at day 17. (**a**) Representative immunoblots (n = 1/group) and expression profile (n = 6/group) of LC3I, LC3II at d17 (Supplemental Fig. S8) and (**b**) representative immunoblots (n = 1/group) and expression profile (n = 6/group) of DNM1 at d17 in the hypothalamus of Control mice (open bars) or mice with limitation of food access (LFA, grey bars) or activity-based anorexia (ABA, black bars). (**c**) Representative immunoblots (from 3 individuals/group) and expression profile (n = 6/group) of BNIP3 and NDP52 at d17 in the hypothalamus of Control mice (open bars) or mice with limitation of food access (LFA, grey bars) or activity-based anorexia (ABA, black bars). *p < 0.05 *vs* Control. ^#^p < 0.05 *vs* LFA.

To determine the activation of autophagy at d17, we performed western blots directed against autophagy markers LC3II and LC3I. As shown in Fig. 7a, LC3I expression was significantly decreased in ABA group (p < 0.05), whereas LC3II had a tendency to increase. Consequently, LC3II/ LC3I ratio, commonly used as a marker of autophagy, was significantly upregulated in ABA mice compared with Controls (p < 0.05). We also observed a trend for an increase in ABA compared with LFA but difference did not reach significance (p = 0.06; Fig. 7a). In addition, BNIP3 expression, a marker of mitophagy, was increased in ABA mice compared with Controls (Fig. 7c). NDP52, another factor involved in mitophagy, was not significantly affected (Fig. 7c). At d17, proteomic analysis also revealed that Dynamin-1 (DNM1) was enhanced in ABA mice (Fig. 3). DNM1 plays a key role in early endosome formation leading to autophagolysosome formation. We confirmed the increase of DNM1 by western blot at d17 (Fig. 7b). All these data showed that autophagy, particularly mitophagy, was increased in the hypothalamus of ABA mice.

### Increased protein synthesis rate in ABA model at d17

**Figure 8:**
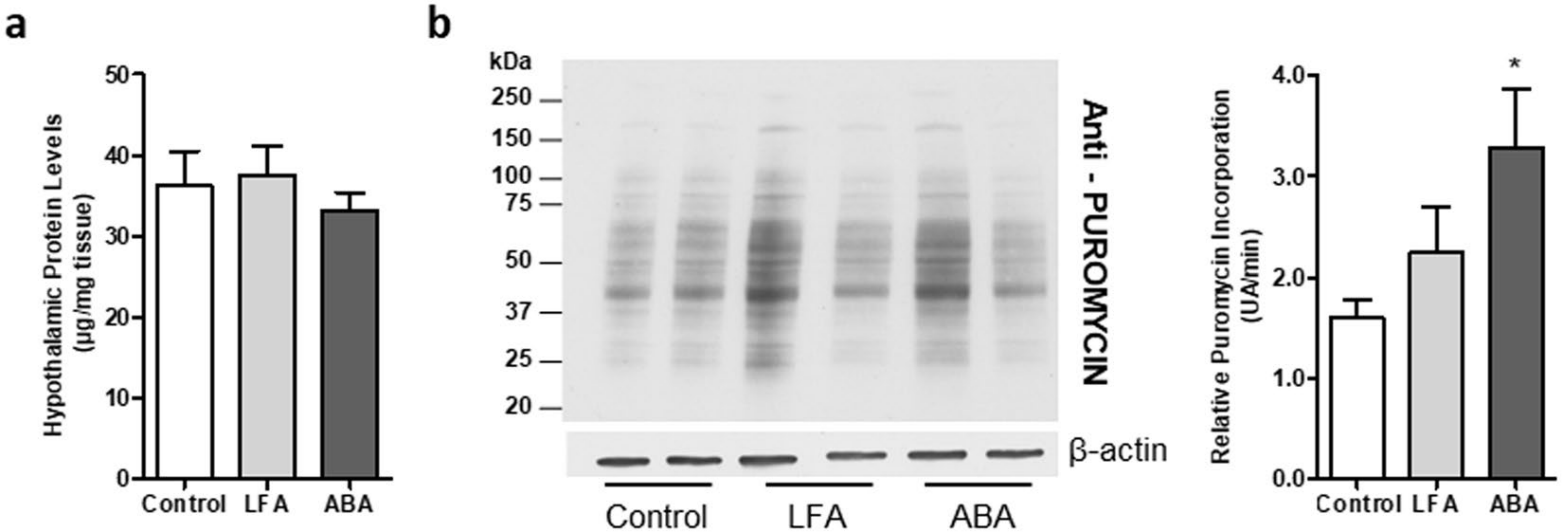
Assessment of protein synthesis using the SUnSET method at day 17. (**a**) Hypothalamic protein content assessed at day 17 in the hypothalamus of Control mice (open bars) or mice with limitation of food access (LFA, grey bars) or activity-based anorexia (ABA, black bars), n = 8/group. (**b**) Representative immunoblots for puromycin and β-actin at day 17 (from 2 individuals/group). Relative puromycin incorporation was expressed as the ratio between puromycin densitometry and time from puromycin injection to tissue sampling (n = 8/group). *p < 0.05 *vs* Control.

Despite the presence of hypothalamic autophagy in ABA mice, hypothalamic protein levels were similar in the three groups (Fig. 8a). We thus evaluate protein synthesis by SUnSET method. Puromycin incorporation significantly increased in ABA group compared with Control group (p < 0.05; Fig. 8b).

Thus, proteomic analysis revealed an adaptive hypothalamic protein response in cachectic ABA female mice with increased protein synthesis, activated autophagy and altered mitochondrial dynamic.

## Discussion

In the present study, we showed that the severe body weight loss induced in the activity-based anorexia model in female C57BL/6 mice is associated in the hypothalamus (i) with altered proteome suggesting an increased energy metabolism, (ii) with altered mitochondrial dynamic and (iii) with an enhanced protein metabolism. Interestingly, ABA mice exhibited a marked body weight loss due to a reduction of both fat and fat-free mass, a reduced spontaneous cumulative food intake compared with Control group. In addition, protein metabolism is altered in soleus muscle of ABA mice with reduced protein synthesis and activated autophagy whereas no modification is observed in anterior tibialis. Accordingly, Enoki *et al*. recently showed in cachectic mice with chronic kidney disease a reduced mass of soleus but no change of anterior tibialis^32^. Finally, in a previous work, we showed the presence of an inflammatory state in ABA mice^30^. All these data suggest that ABA model can be considered as a model of anorexia cachexia syndrome.

In female ABA mice, our results showed alterations of hypothalamic proteome, in particular of several proteins involved in glycolysis or in TCA cycle suggesting an enhanced energy metabolism in the hypothalamus. An increased hypothalamic neuronal activity has been previously reported in different area of hypothalamus (arcuate nucleus, dorsomedial and lateral hypothalamus) during the ABA model in rats by c-fos immunostaining^33^. Interestingly, both at d10 and d17, modified proteins were mainly involved in metabolic pathways: glycolysis and TCA cycle. Indeed, we observed an increase of GAPDH, ENO2 and PDHB expression in ABA mice, enzymes involved in the glycolysis leading to Acetyl-CoA availability in the mitochondria for TCA cycle. In addition, ACLY, ACO2 and OGDH overexpression also suggest that energy production was enhanced in the hypothalamus of ABA mice. Glyoxalase domain-containing protein 4 (GLOD4) and Peptidyl-prolyl cis-trans isomerase (FKBP4) were also modulated in hypothalamus of ABA mice, respectively at d10 and d17, while these proteins contribute to limit oxidative stress^34,35^. Many studies reported overexpression of NPY in the arcuate nucleus in response to negative energy balance or food restriction^36,37^, that contributes to the increase of hypothalamic activity. Interestingly, increased activity of NPY-AgRP neurons during negative energy balance was associated with an increase of mitochondrial fission^38^. Raefsky & Mattson recently reviewed mitochondrial and energy metabolism responses after different stress including exercise and fasting and they concluded that dietary energy restriction is associated with mitochondria biogenesis in brain at least at short-term^39^ In our study, enhanced expression of mitochondrial proteins can be related to an increase of hypothalamic energy needs to activate orexigenic pathways but also to an increase of mitochondria number. Our proteomic analysis revealed a protein involved in the regulation of mitochondrial fission named DNM1L (also called DRP1)^40^. DNM1L is active for mitochondria fission only in its phosphorylated form. By immunoblotting, we observed that p-DNM1L/DNM1L ratio was only increased at d17 whereas total DNM1L expression was enhanced from d10, suggesting an increase of mitochondria number through mitochondrial fission activation. By contrast, markers of mitochondrial fusion, mitofusins and OPA1, and of mitochondrial biogenesis, PGC-1α and TFAM, remained unchanged. We were not able to evaluate mitochondria number. Citrate synthase activity and VDAC1 immunostaining, markers of mitochondrial membrane surface, were also not affected at d17 in ABA mice. In accordance with our results, Dietrich *et al*. reported that a short-term food restriction (24 h) was associated to an activation of mitochondrial fission without affecting mitochondrial fusion in AgRP neurons leading to an increased number of mitochondria that however exhibited a lower size^20^. Conversely, alteration of mitochondrial dynamic in POMC expressing neurons by either mitofusin 2^19^ or DMN1L^18^ deficiencies modified food intake and whole body energy homeostasis. All these data clearly show that mitochondrial dynamic is closely linked to feeding behaviour. However, to our knowledge, there were no studies evaluating the effects of a long-term food restriction before the present study on hypothalamic mitochondrial dynamic.

Interestingly, Kaushik *et al*. provided evidence that short-term food restriction (6 h) induced autophagy in hypothalamic AgRP neurons contributing to higher expression of AgRP^17^. Whether mitochondria are directed or not towards autophagy could explained the discrepancy observed in the present work between activation of mitochondrial fission and unchanged mitochondrial membrane surface. After a long-term negative energy balance, mitochondria may be addressed to mitophagy. We thus analyzed autophagy by evaluating LC3II/LC3I ratio commonly used as a marker of autophagosome formation^41^. LC3II/LC3I ratio was markedly increased in ABA group compared with Control group, as well as BNIP3, a marker of mitophagy. Accordingly, proteomic analysis also revealed that DNM1 expression was increased in ABA mice. DNM1 acts on synaptic vesicle endocytosis leading to lysosome formation and hence autolysosome formation with the fusion of autophagosome^41^. Autophagy is associated with modifications of cytoskeleton^42^, and in the present study, we identified two altered proteins involved in cellular component organization: Alpha-internexin (INA) and Tropomyosin alpha-3 chain (TPM3). Thus, our data show that ABA mice exhibited activation of autophagy in the hypothalamus that could be the consequence of the severe weight loss associated with reduced food intake (13.6% of body weight loss). In male mice with short-term fasting, an up-regulation of neuronal autophagy has been reported in the cortex^43^. We thus speculate that activation of autophagy blunted the increase of mitochondria number. Activation of autophagy is considered as a protective process during food restriction at least at short term to provide energy to cells but autophagy has also been proposed to stimulate food intake through NPY and POMC regulation^44^. By contrast, NPY has also been shown to induce autophagy in the hypothalamus^45^. Mice with autophagy deficiency in the hypothalamus show different phenotypes accordingly to the site of deficiency and used methods^46^ but all these data confirm that autophagy is involved in the control of food intake and energy metabolism. The role of induced autophagy/mitophagy in the hypothalamus of ABA female mice should be thus further studied, as well as the autophagic flux by combining autophagy probes and flux cytometry as previously described^47^.

Despite hypothalamic autophagy, we did not observe a decrease of hypothalamic protein content in ABA mice compared with other groups. For this reason, we assessed hypothalamic protein synthesis with SUnSET method which employs puromycin as a nonradioactive label^48^. We clearly observed an increase of hypothalamic protein synthesis rate in ABA mice. To our knowledge, we thus demonstrated for the first time that protein synthesis is markedly increased in the hypothalamus of severely undernourished mice. In the present study, we did not evaluate activities of other proteolytic pathways, i.e. proteasome or calpains, that should be further studied.

In the liver, it has been recently showed that mitochondrial morphology and mitochondrial autophagy were regulated by the circadian clock system^49^. In physiological conditions, diurnal rhythm coincided with feeding/fasting cycle and is associated with mitochondrial remodelling. In the present study, mice have been euthanized at the beginning of the dark phase before food distribution, we cannot exclude that mitochondrial metabolism may be different during the light phase. Similarly, increased hypothalamic energy and mitochondrial metabolism may be the consequence of food anticipatory activity. In the same way, we observed that AMPK was less activated in the hypothalamus of LFA and ABA mice while other studies previously showed activation of AMPK after food deprivation^50,51^. However, hypothalamic AMPK can be affected by stress, i.e. social defeat stress^52^ or by circadian rhythm^53,54^, two factors involved in ABA model. The effect of physical activity alone on hypothalamic proteome in mice with ad libitum access to food should be also of interest, even if physical activity pattern was changed in ABA mice^55,56^.

Finally, DNM1 is also involved in the regulation of internalization of G-protein receptor, i.e. melanocortin-4 receptor (MC4R) internalization. Even if we were not able in the present study to evaluate MC4R localization in the hypothalamus and most importantly its subcellular localization, we can speculate that MC4R defect in ABA mice by increasing its internalization may be an adaptative response to the weight loss as previously reported^21,57^.

Further studies should be done to better understand the role of these hypothalamic alterations in the control of food intake and energy homeostasis. Particularly, it should be of interest to better characterize hypothalamic nuclei and cell types that exhibit those alterations, to evaluate the role of circadian rhythm on hypothalamic mitochondrial dynamic and to know whether these mechanisms may affect ghrelin or leptin sensitivity.

In conclusion, by using the activity-based anorexia model, we showed that hypothalamic proteome is altered during severe weight loss associated with reduced food intake. Most interestingly, activity-based anorexia is associated with an increased energy metabolism, modifications of mitochondrial dynamic and an induction of mitophagy in the hypothalamus.

## Methods

Authors confirm that all experiments were performed in accordance with relevant guidelines and regulations (Official Journal of the European Community L 358, 18/12/1986). M.C. was authorized by the French government (authorization no. 76-107). Furthermore, the protocol was approved by the local ethical committee named CENOMEXA (authorization on N/05-11-12/28/11-15).

### Animal protocols

Female 8-week-old C57BL/6 mice (Janvier Labs focusing system (GE Healthcare) weighing approximately 18-19 g were acclimatized one week at 23 °C with a reversed 12-hour light-dark cycle (dark phase: 10:30 AM – 10:30 PM) and then were randomized into 3 groups: an *activity-based anorexia* group (ABA, n = 16), a *limited-food access* group (LFA, n = 16) and an *ad libitum* group (Control, n = 16). ABA procedure was performed as previously described^28–30^. Briefly, ABA mice were placed in cages with an activity wheel connected to Running Wheel® software (Intellibio, Seichamps, France), while both LFA and Control mice were placed in standard cages. From day 6, LFA and ABA mice had a progressive limited access to food from 6 h/day at day 6 to 3 h/day at day 9 and until the end of experiment. Food access was given at the beginning of the dark phase (10:30 AM). All mice had free access to water. For ethical reasons, if weight loss exceeded 20% during 3 consecutive days, mice were euthanized. At the end of experiment, mice were injected with lethal dose of ketamine/largactil (40 and 1 mg.kg^−1^, respectively) at day 10 (n = 8/group) or at day 17 (n = 8/group) corresponding to an initial and a persistent weight loss, respectively. All mice were sacrificed at the beginning of the dark phase before food distribution. Brain was immediately removed and hypothalamus was taken and stored at −80 °C until analysis.

A second experiment was performed with C57BL/6 female mice to measure protein synthesis rate. Mice were randomized into 3 groups produced as described before: ABA (n = 8), LFA (n = 8) and Control (n = 8). Body weight, activity wheel, food and water intake were daily monitored. At day 17, mice were given an intraperitoneal injection of 0.040 µmol/g puromycin (Sigma Aldrich, Saint Quentin Fallavier, France) dissolved in 100 µl of PBS and then were euthanized 30 min after injection. Hypothalamus and muscles (soleus and tibialis anterior) were rapidly removed and frozen in liquid nitrogen^48^.

A third experiment performed in C57BL/6 female mice was performed in LFA (n = 8) and ABA (n = 8) mice to evaluate 1 hour – food intake. The ABA and LFA procedures were performed as described above. At day 17 (10:30 AM), mice were placed in standard cages without activity wheel and had free access to food during 1 hour.

### Body composition

Whole body composition were assessed as previously described^29^ at day 17 by using EchoMRI™ EMR-185 (EchoMRI, Houston, TX), a quantitative nuclear magnetic resonance system for vigil mice (n = 8/group).

### Protein extraction for a two-dimensional gel electrophoresis (2-DE)

Hypothalamus were homogenized, as previously described^58^, in ice-cold lysis buffer containing 7 mol/L urea, 2 mol/L thiourea, 4% (w/v) CHAPS, 25 mmol/L spermine tetrahydrochloride, 50 mmol/L dithiothreitol, 0.5% (v/v) immobilized pH buffer pH 3–10 non-linear (GE Healthcare, Velizy-Villacoublay, France), and a protease inhibitor cocktail (Sigma Aldrich). The protein content was determined by using 2D Quant kit (GE Healthcare).

Total proteins (40 µg for silver staining or 400 µg for colloidal Coomassie Blue G-250 staining) were included in a rehydration solution and were taken up into pH 3–10 nonlinear gradient Immobiline DryStrip (GE Healthcare) during 12 h. Then, IEF was performed with the current limit at 50 mA per strip and with a total of 8,000 V-h using the IPGphor isoelectric. After focusing, IPG strips were equilibrated for 30 min in the equilibration buffer containing 6 mol/L urea, 30% (v/v) glycerol, 2% (w/v) sodium dodecyl sulphate, 50 mmol/L Tris-HCl pH 8.8 with 2% (w/v) dithiothreitol. Then strips were alkylated for 20 min in the same equilibration buffer containing 4% (w/v) iodoacetamide instead of dithiothreitol and 0.25% (w/v) bromophenol blue. After equilibration, IPG strips were affixed onto 8–16% polyacrylamide gradient gels (20 cm × 18 cm × 1 mm) for SDS-PAGE. The second dimension was performed overnight in the Ettan Daltsix vertical system (GE Healthcare) at 18 mA per gel. After SDS-PAGE, the 2D gels were stained with silver nitrate method (PlusOne Silver Staining kit; GE Healthcare) for gel analysis or dyed following the colloidal Coomassie Blue G-250 staining method for mass spectrometric analysis^59^. Two-dimensional gel electrophoresis was performed in triplicate for six samples per group (n = 6/group at each time) to minimise variations in migration, and each set of 3 gels was subjected to image analysis to confirm the nonappearance of statistically differential spots within the set of gels.

### Analysis and comparisons of 2D gel images

Silver stained gels were scanned with an ImageScanner II (GE Healthcare) previously calibrated by a greyscale marker (Kodak) and 2D images were captured with Labscan 6.00 software (GE Healthcare). Differential analysis was realized by using Progenesis SameSpots v4.1 software (Nonlinear Dynamics Ltd, Newcastle, U.K.) for spot detection, background subtraction, spot volume normalisation and expression. The most representative silver-staining gels (gel migration, spot definition, and spot number) of each sample (n = 6 per group) was used to identify differential hypothalamic protein expression. The expression level was determined by dividing the raw quantity of each spot by the total intensity value of all the pixels in the image, and expressed as % volume, corresponding to spot volume/∑volumes of all spots in gel. Variations in abundance, corresponding to normalised spot volume, were calculated as the ratio of average values of % volume for each group. Only spots with a % volume variation >1.4 were included in the analysis. The absence of a spot within a gel indicated that detectable expression matches the background of gel. Statistical differences between the groups were determined using ANOVA (significance level p values < 0.05).

### In-gel digestion for mass spectrometry

Protein spots of interest were manually excised from colloidal Coomassie blue-stained 2D gels and digested. Briefly, gel fragments were washed three times for 20 min in destain solution containing 50% (v/v) methanol and 50 mmol ammonium bicarbonate/L. The excised spots were then air-dried for 1 h, which was followed by in-gel digestion in 30 µL of a digestion buffer containing 50 mmol/L ammonium bicarbonate and 6 ng/µL sequencing grade bovine trypsin (Roche Diagnostics, Meylan, France). After overnight digestion, the aliquots were treated with 50% (v/v) acetonitrile and 5% (v/v) formic acid. Speed-vas-dried peptide extracts were resuspended in 10 µL of 5% (v/v) acetonitrile and 0.1% (v/v) formic acid and then analyzed with a nanoflow liquid chromatograph LC1200 system coupled to a Q-TOF 6520 or to a 6340 Ion Trap mass spectrometer equipped with a nanospray source and a HPLC-chip cube interface (Agilent Technologies, Les Ulis, France). Briefly, peptides were enriched and desalted on a 40 nL C18 reversed-phase trap column and separated on a Zorbax C18 column (75 µm inner diameter x 43 mm long, 5 µm particle size and 30 nm pore size; Agilent Technologies). Peptides are eluted at 400 nL/min using a 17 min (for Q-TOF) or 9 min (for Ion Trap) linear gradient (3–80% acetonitrile in 0.1% formic acid). Then, the eluent was analyzed with a Q-TOF or Ion Trap mass spectrometer.

### Protein identification by LC-ESI-MS/MS

For protein identification, MS/MS peak list were extracted and compared with MASCOT Daemon version 2.2.2 (Matrix Science, Boston, MA) server, using the following parameters: enzyme specificity, trypsin; no restriction on protein molecular weight; one missed cleavage permitted; no fixed modification; variable modifications, methionine oxidation, cysteine carbamidomethylation, serine, tyrosine, and threonine phosphorylation; monoisotopic; peptide charge, 2+ and 3+; mass tolerance for peptide, ±20 ppm (Q-TOF) or ±1,5 Da (Ion Trap); mass tolerance for fragment ions, ±0.06 Da (Q-TOF) or ±0.5 Da (Ion Trap); ESI-QUAD-TOF and/or ESI-TRAP as instrument; taxonomy, mus musculus; database, UniProtKB/Swiss-Prot. Protein matching probabilities were validated if proteins hits satisfied one of following criteria: MASCOT score of >32 (p < 0.01) or >22 (p < 0.05) with identification confidence ≥3 top-ranking peptides (number of matching/the coverage of protein sequence). False discovery rate (FDR) assessment was determined using the “decoy” option of MASCOT (target database, SwissProt mus musculus). Results were considered relevant if the FDR was 0%.

### Gene ontology (GO)-based analysis

STRING 10.0 software was used as search tool for the retrieval of interacting genes/proteins database. UniProt GO (Biological Process) annotation, PANTHER (Protein ANalysis Through Evolutionary Relationships) classification and KEGG (Kyoto Encyclopedia of Genes and Genomes) Pathway were used to categorise proteins on the basis of their biological process.

### Evaluation of protein expression by western blot

Total proteins (25 µg proteins/lane) were separated on a 4–20% gradient polyacrylamide gel (Biorad, Marnes-la-Coquette, France). After electrophoresis (200 V for 35 min), proteins were transferred onto nitrocellulose membrane (GE Healthcare) by using a semidry transfer method (0.8 mA/cm^2^ for 1h45). After transfer, nitrocellulose membranes were blocked in TBS-T solution (10 mmol Tris-HCl/L pH 8, 150 mmol NaCl/L, 0.2% (v/v) Tween 20) with 5% (w/v) bovine serum albumin (BSA) for 2 h at room temperature. Then, membranes were incubated overnight at 4 °C in TBS-T and 5% (w/v) BSA with specific primary antibodies: goat polyclonal antibodies [anti-dynamin-1 (DNM1, sc-6402, 1:1,000; Santa Cruz Biotechnology, Nanterre, France), and anti-dynamin-1-like protein (DNM1L, sc-21804, 1:1,000; Santa Cruz Biotechnology)], rabbit polyclonal antibodies [anti-Phospho-DNM1L Ser616 (p-DNM1L, 3455, 1:1,000; Cell Signaling Technology, Danvers, MA), anti-LC3B (LC3I/LC3II, NB100-2220, 1:2,000; Novus Biologicals, Littleton, CO), anti-PGC-1α (Peroxisome proliferator-activated receptor coactivator-1 alpha, NBP1-04676, 1:2,500; Novus Biologicals), anti-TFAM (Transcription Factor A, Mitochondrial, ABE483, 1:2,500; Merck Millipore, Molsheim, France), and anti-BNIP3 (BCL2/adenovirus E1B 19 kDa protein-interacting protein 3, #3769, 1:1,000; Cell Signaling Technology/Ozyme, Saint Quentin en Yvelines, France), anti-phospho-AMPKα, (phosphorylated- AMP-activated protein kinase, #2535, 1:1,500; Cell Signaling Technology), anti-AMPKα (#5831, 1:1,500; Cell Signaling Technology), anti-phospho-ACC (acetyl-pphosporylated Acetyl-CoA carboxylase, #11818, 1:1,500; Cell Signaling Technology), anti-ACC (#3676, 1:1,500; Cell Signaling Technology), anti-mitofusin 2 (#9482, 1:2,000; Cell Signaling Technology), anti-OPA1 (optic atrophy 1, #NBP2-59770, 1:1,500; Novus Biologicals)] and mouse polyclonal antibody [anti-NDP52 (nuclear dot protein 52 kDa, H00010241-B01P, 1:1,000; Novus Biologicals), anti-mitofusin 1 (NBP1-71775, #2535, 1:2,000; Novus Biologicals)]. Then, nitrocellulose membranes were washed three times for 5 min with TBS-T, followed by incubation with horseradish peroxidase-conjugated antibodies (all 1:5,000) [swine polyclonal anti-rabbit IgG-HRP (P0399; Dako, Trappes, France), donkey anti-goat IgG-HRP (sc-2020; Santa Cruz Biotechnology), rabbit polyclonal anti-mouse IgG-HRP (P0161; Dako)] for 1 h at room temperature. After three washes for 5 min in TBS-T, immunocomplexes were revealed by using the ECL detection system (GE Healthcare). The blots were stripped and reblotted with a mouse monoclonal antibody anti-β-actin (A5441, 1:5,000; Sigma Aldrich) also used as a loading control. Protein bands were quantified by using an ImageScanner III densitometer (GE Healthcare) and ImageQuant TL software (GE Healthcare).

### Evaluation of mitochondrial morphology by measure of citrate synthase activity

The Citrate Synthase (CS) Activity Assay kit (MAK193; Sigma Aldrich) has provided a direct procedure for measuring the activity of CS in hypothalamus of mice. For total protein extraction, hypothalamus sections were homogenized at 4 °C in lysis buffer and the protein content was determined by using Bradford assay. The assay contained 12 µl total protein per well (all samples has been run in duplicate). The A412 readings were taken on a ∑ 960 plate reader (Metertech, Taipei, Taïwan) with 5 readings over the 2.0 min time span. These A412 readings were located within the linear range of the standard curve. We used the formula provided by the manufacturer to calculate total CS activity.

### Immunostaining of mitochondria

Brain sections were embedded in Tissue-Tek (OCT compound, Fisher Scientific) and immediately frozen at −80 °C. Then, sections of frozen brain (10 µm thick) were performed using a cryostat (Leica Microsystems) and were mounted on glass slides, and air dried. After a step of fixation with 4% of paraformaldehyde and of permeabilization with a mixture of PBS/0.2% triton X-100, nonspecific binding was blocked with PBS containing 0.1% bovine serum albumin (BSA, Sigma Aldrich), 5% non-immune goat serum (NGS) and 0.05% tween 20 for 20 min at room temperature. Then, the sections were incubated at 4 °C overnight in the same solution supplemented with primary antibody, anti-Voltage-dependent anion-selective channel 1 (anti-VDAC1, Novus biologicals, Littleton, CO). After 3 washes with PBS, sections were incubated with FITC-conjugated secondary Antibodies (LifeTechnologies) for 1h30 at room temperature in the dark. After 3 washes with PBS, cover-slips were mounted with a drop of mounting medium for fluorescence with DAPI (Vector Laboratories, Peterborough, UK). After immunohistochemistry, microphotographs were acquired with DM5500 B fluorescence microscope equipped with a Photometrics CoolSNAP HQ2 camera (Leica) for detection of DAPI or FITC with 10× and 63× objectives.

### Evaluation of protein synthesis by SUnSET method

Surface sensing of translation (SUnSET) is a nonradioactive method for *in vivo* measurement of protein synthesis^60^. Puromycin-conjugated peptides were visualized by western blotting as described above by using mouse monoclonal anti-puromycin antibody (MABE343, clone 12D10, 1:5,000; Merck Millipore, Darmstadt, Germany). The density of each whole lane has been then measured to determine the protein synthesis rate expressed as puromycin relative expression/min.

### Statistical analysis

The data are expressed as mean ± standard error mean (SEM). Comparisons between two groups were assessed using the unpaired two-tailed Student’s t-test or non-parametric Mann-Whitney test. Differences between more than two groups were performed using one-way ANOVA test followed by Dunnet’s multiple comparisons as *post hoc* tests or by two-way ANOVA followed by Bonferroni’s post-tests, as appropriate. All calculations were realized by using GraphPad Prism 5.0 (GraphPad Software Inc.) and p < 0.05 was considered significant.

## Electronic supplementary material

Supplementary Information
