# Supplementary material for: Alterations of proteome, mitochondrial dynamic and autophagy in the hypothalamus during activity-based anorexia
Source: Sci Rep. 2018 May 8;8:7233. doi: 10.1038/s41598-018-25548-9 (PMC5940678; doi:10.1038/s41598-018-25548-9)
Supplement: Supplementary file 1 — Supplementary Information [file 41598_2018_25548_MOESM1_ESM.doc]

**Alterations of proteome, mitochondrial dynamic and autophagy in the hypothalamus during activity-based anorexia.**

Séverine Nobis, Alexis Goichon, Najate Achamrah, Charlène Guérin, Saida Azhar, Philippe Chan, Aline Morin, Christine Bôle-Feysot, Jean Claude do Rego, David Vaudry, Pierre Déchelotte, Liliana Belmonte, Moïse Coëffier

**a**


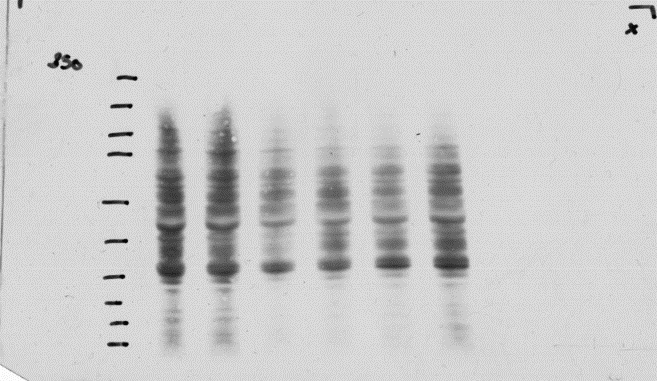


**Anti - PUROMYCIN**


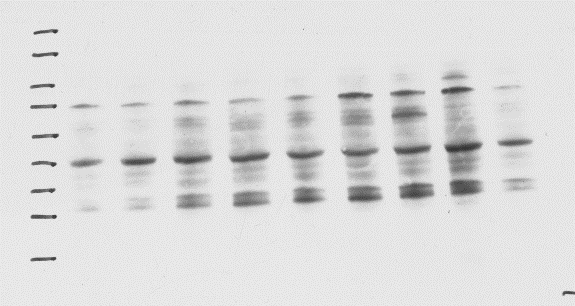


**b**

**Anti - PUROMYCIN**

**c**

**d**


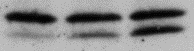


**LC3I**

**LC3II**

**LC3I**

**LC3II**


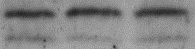


***soleus***

***tibialis***

**Supplemental Fig. S1: Muscle protein metabolism**

Representative immunoblots and densitometric analysis for puromycin (**a.** and **b.**) and LC3II / LC3I (**c.** and **d.**) at day 17 in soleus (**a.** and **c.**) and anterior tibialis (**b.** and **d.**) from Control mice (open bars) or mice with limitation of food access (LFA, grey bars) or activity-based anorexia (ABA, black bars). *, p < 0.05 *vs* Control (n=8 /group).

**Supplemental Fig. S1: Muscle protein metabolism**

Representative immunoblots and densitometric analysis for puromycin (**a.** and **b.**) and LC3II / LC3I (**c.** and **d.**) at day 17 in soleus (**a.** and **c.**) and anterior tibialis (**b.** and **d.**) from Control mice (open bars) or mice with limitation of food access (LFA, grey bars) or activity-based anorexia (ABA, black bars). *, p < 0.05 *vs* Control (n=8 /group).

**Supplemental Fig. S2: 1h-Food intake in LFA and ABA mice at day 17**

Food intake measured during the first hour at day 17 in mice with limitation of food access (LFA, grey bars) or activity-based anorexia (ABA, black bars). *, p < 0.05 *vs* LFA (n=6 / group).

**Supplemental Fig. S3:** Representative silver-stained gel images from Control, LFA and ABA mice.

Differentially expressed proteins (> 1.4-fold change; circled spots with a letter) were determined by statistical analysis (ANOVA one-way, p < 0.05). Protein spots identification is displayed in Table 1.


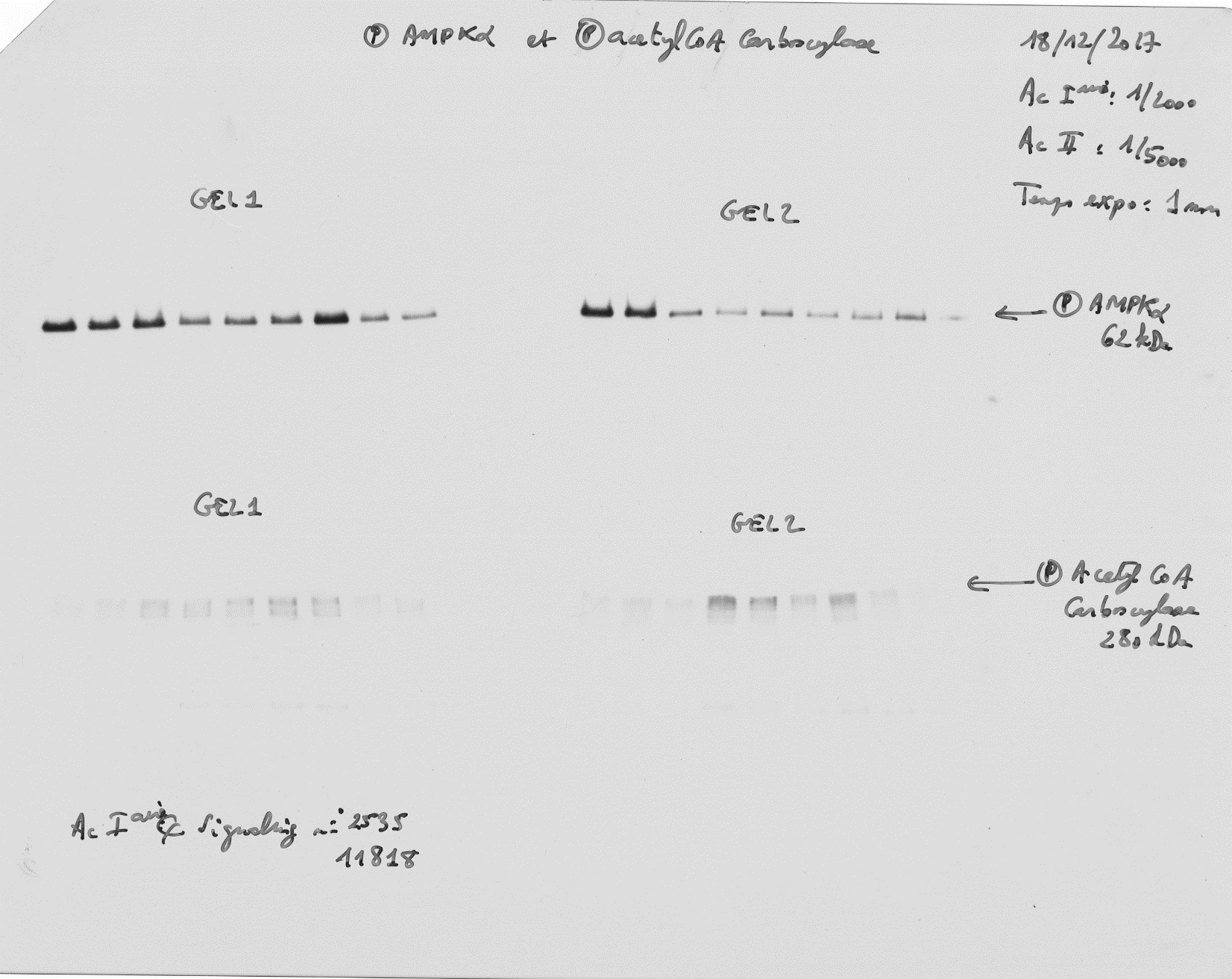

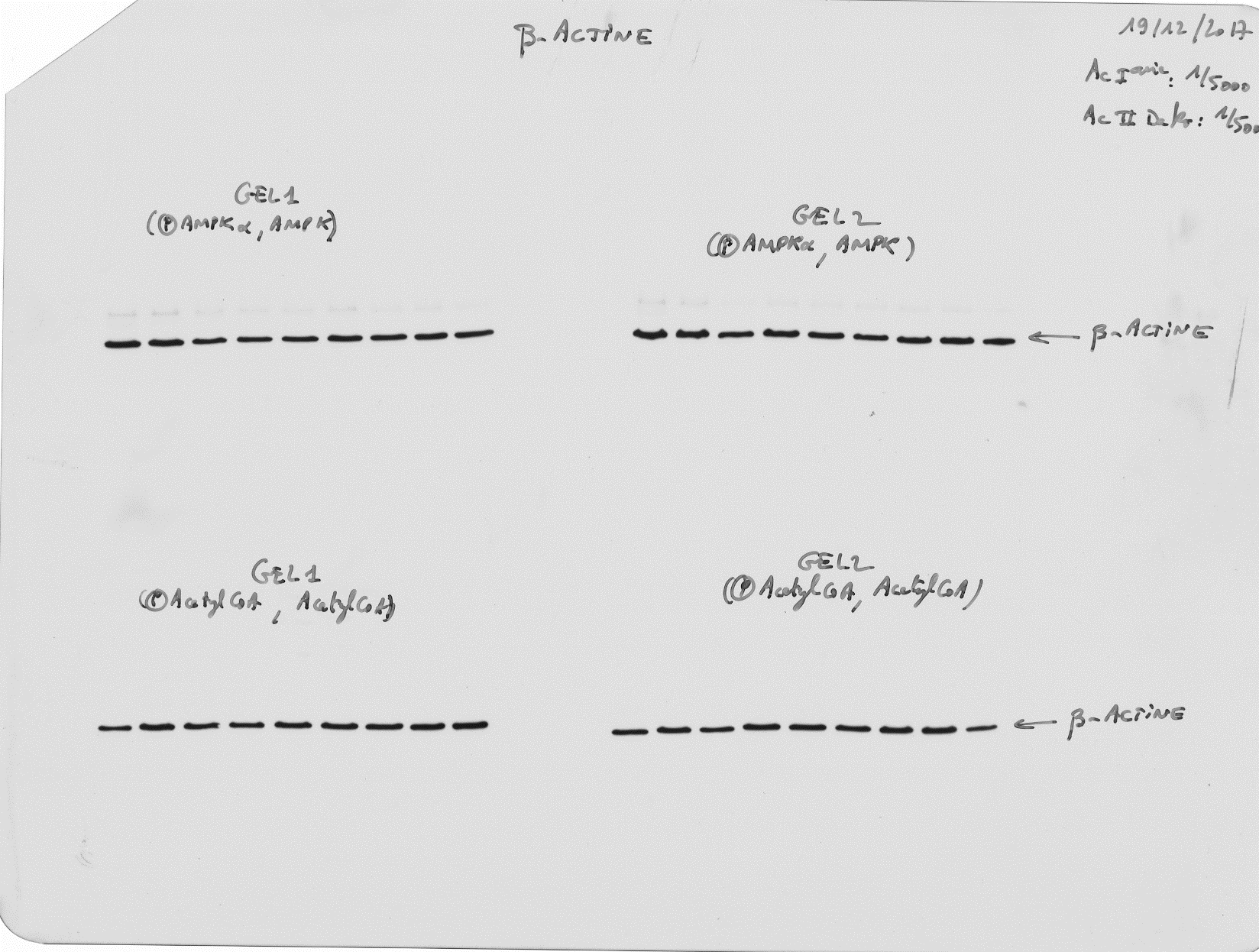

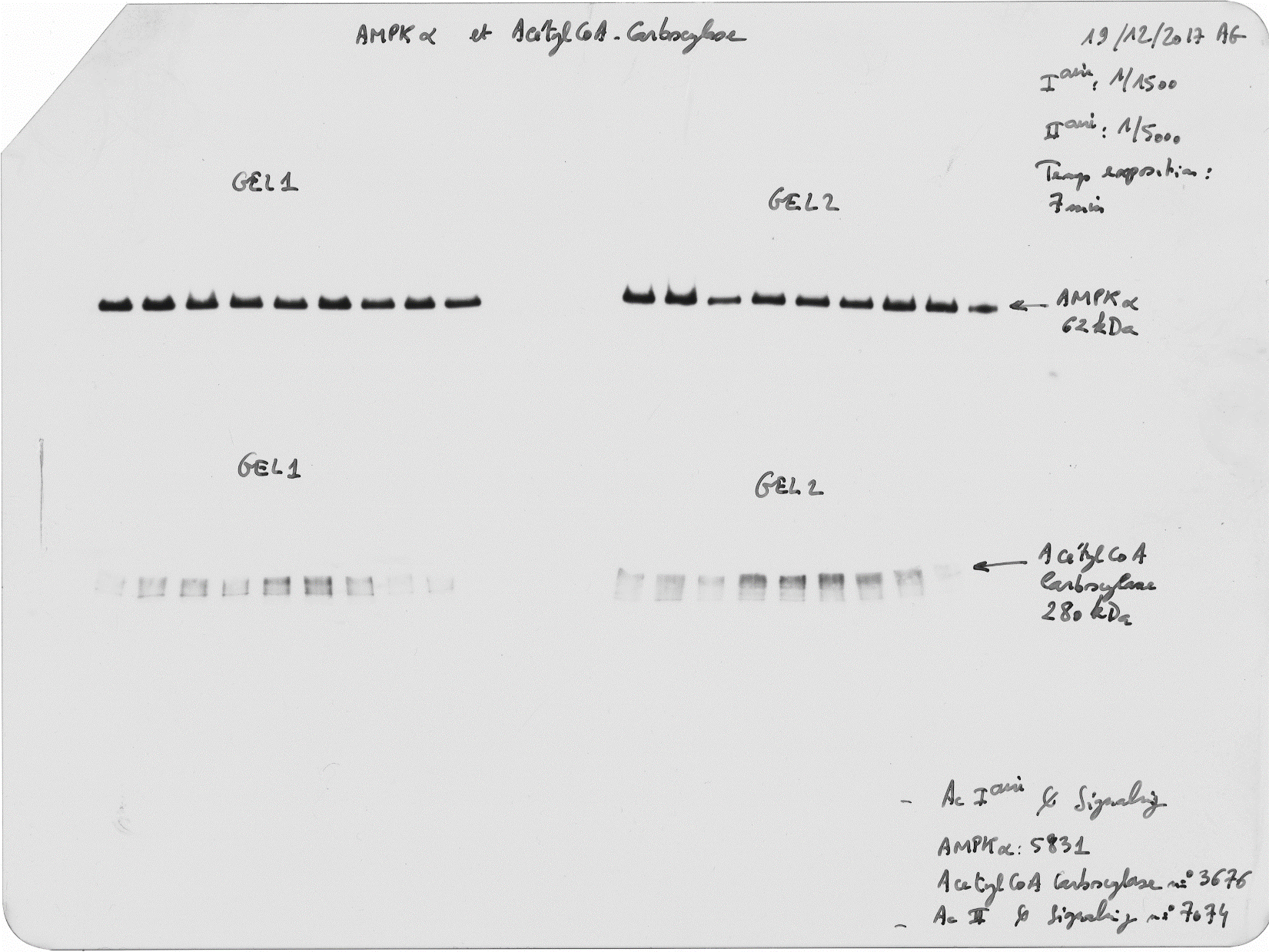

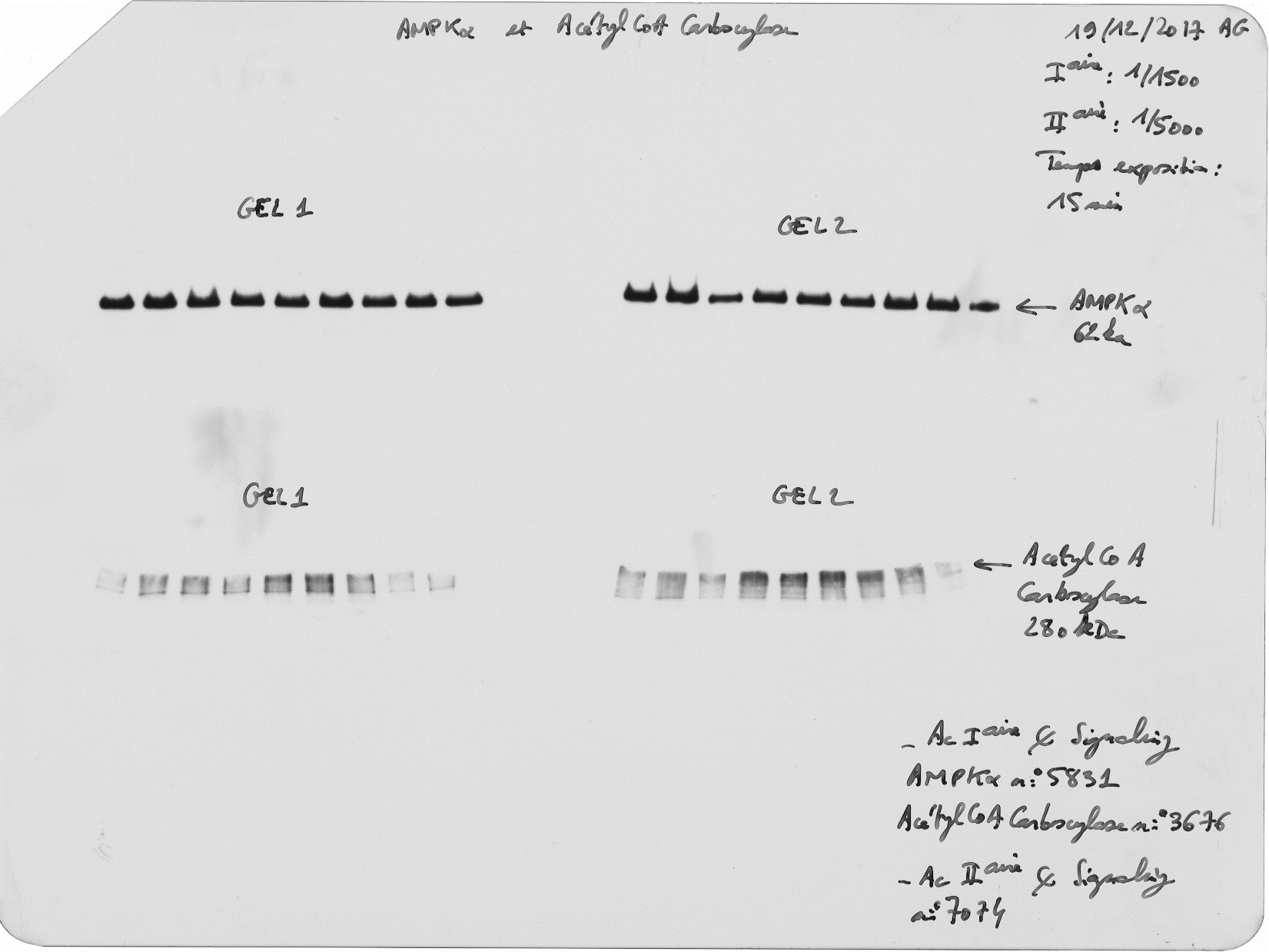

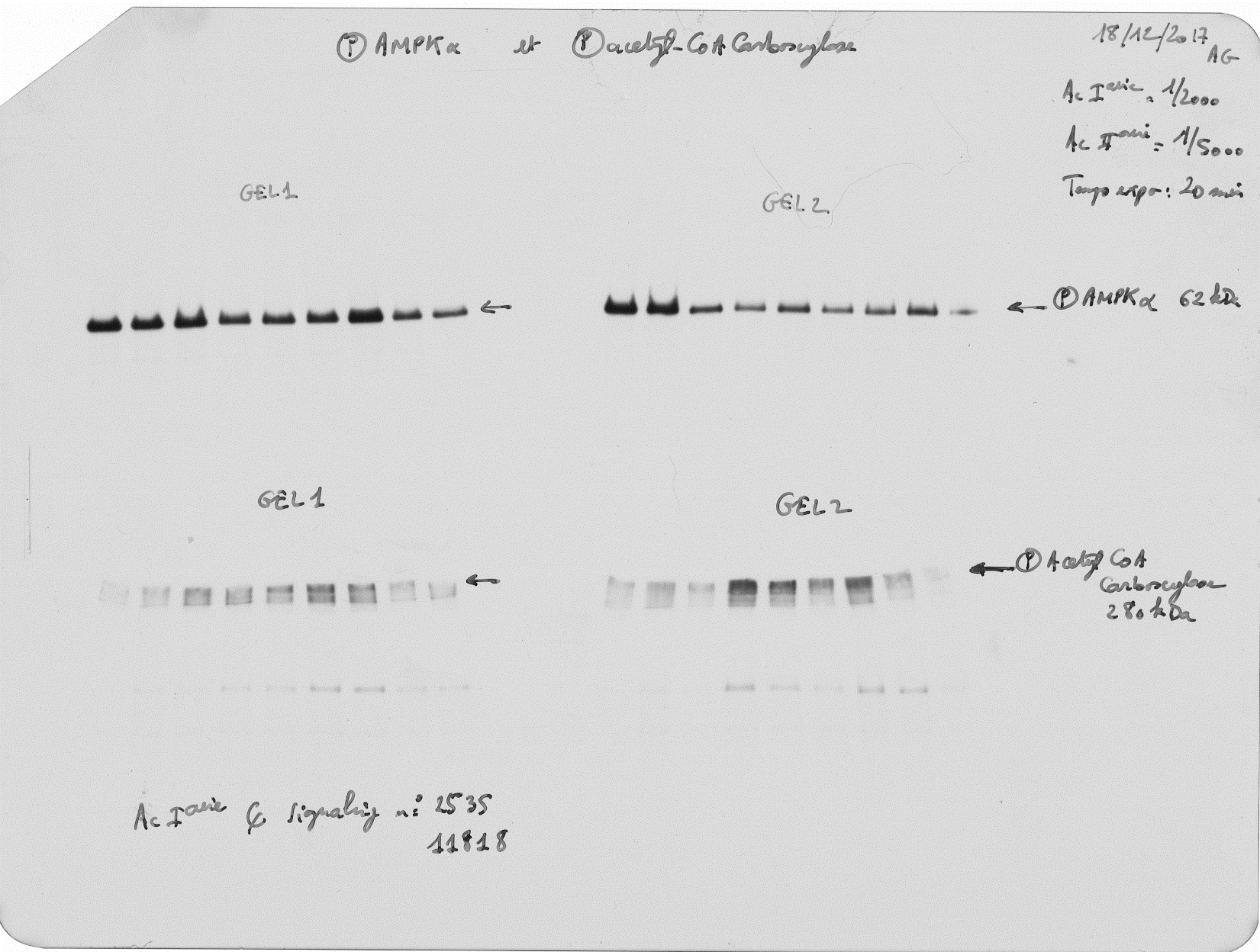


p-AMPKα

AMPKα

p-ACC

ACC

β-actin

**Control**

**LFA**

**ABA**

**Supplemental Fig. S4: Immunoblots for AMPK and ACC in the hypothalamus.**

Representative immunoblots and densitometric analyses of AMPK, ACC and β-actin at day 17 in the hypothalamus obtained from Control mice or mice with limitation of food access (LFA) or activity-based anorexia (ABA). (from 6 individuals /group). *, p < 0.05 vs Control.


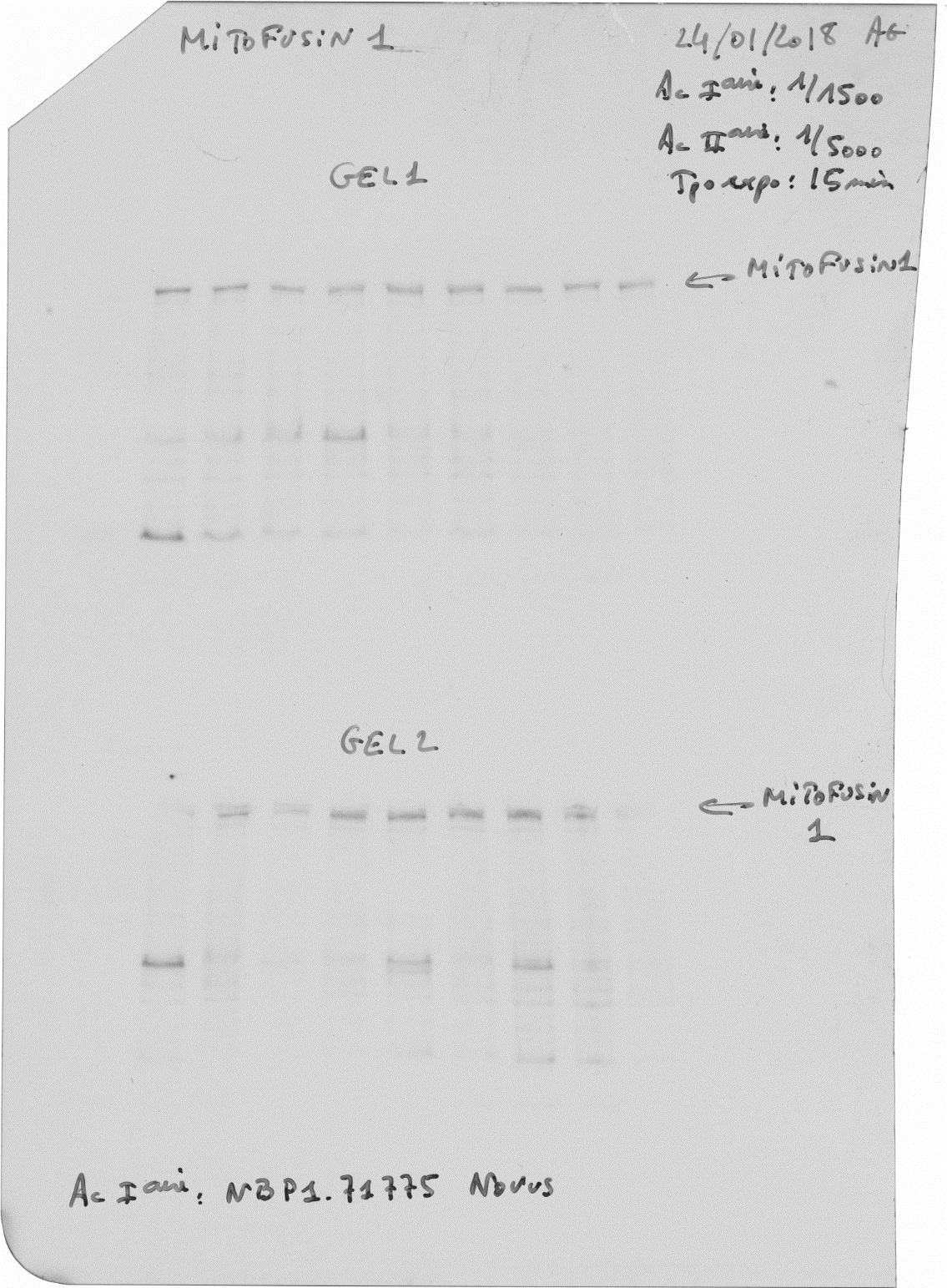

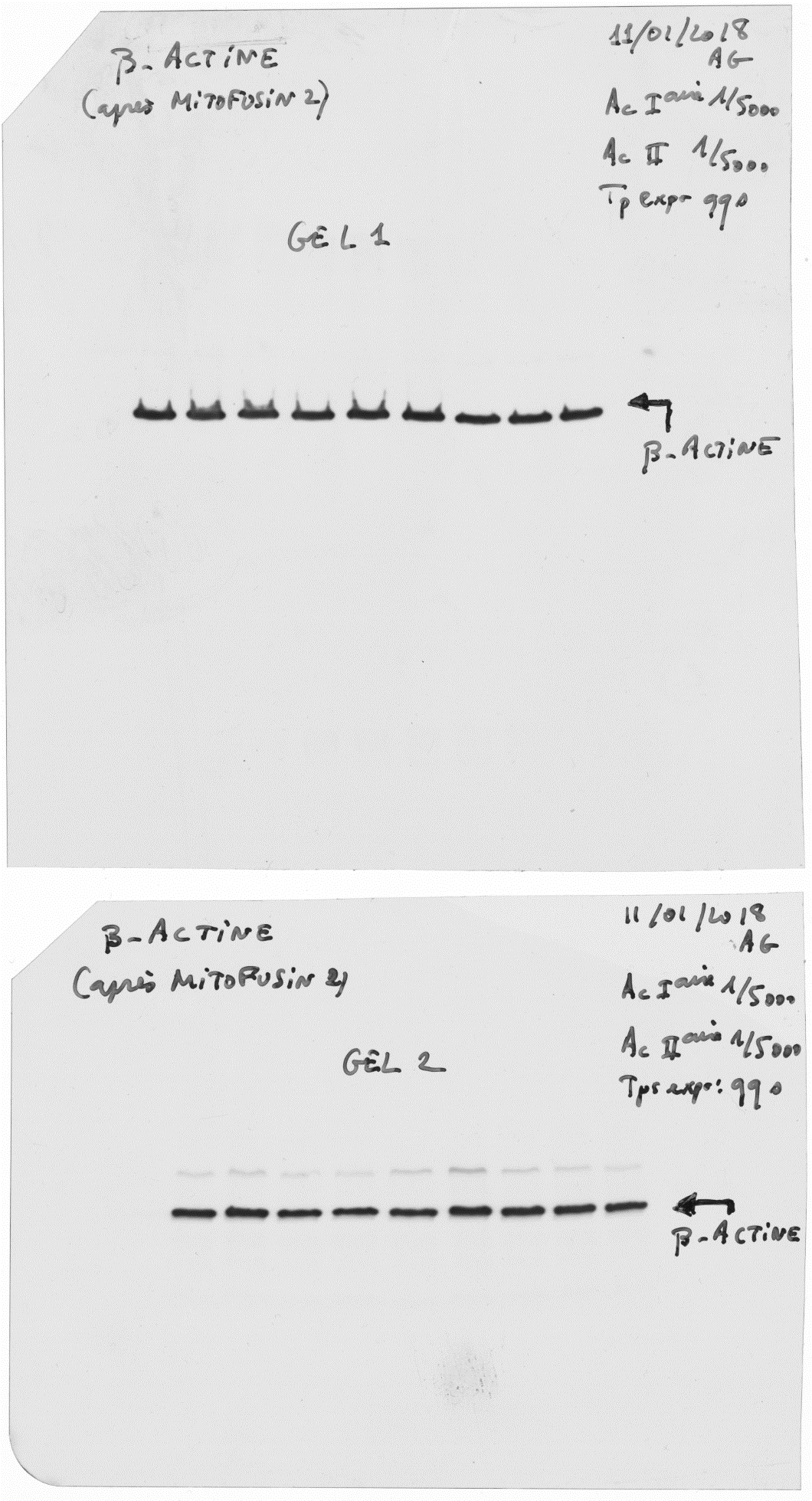

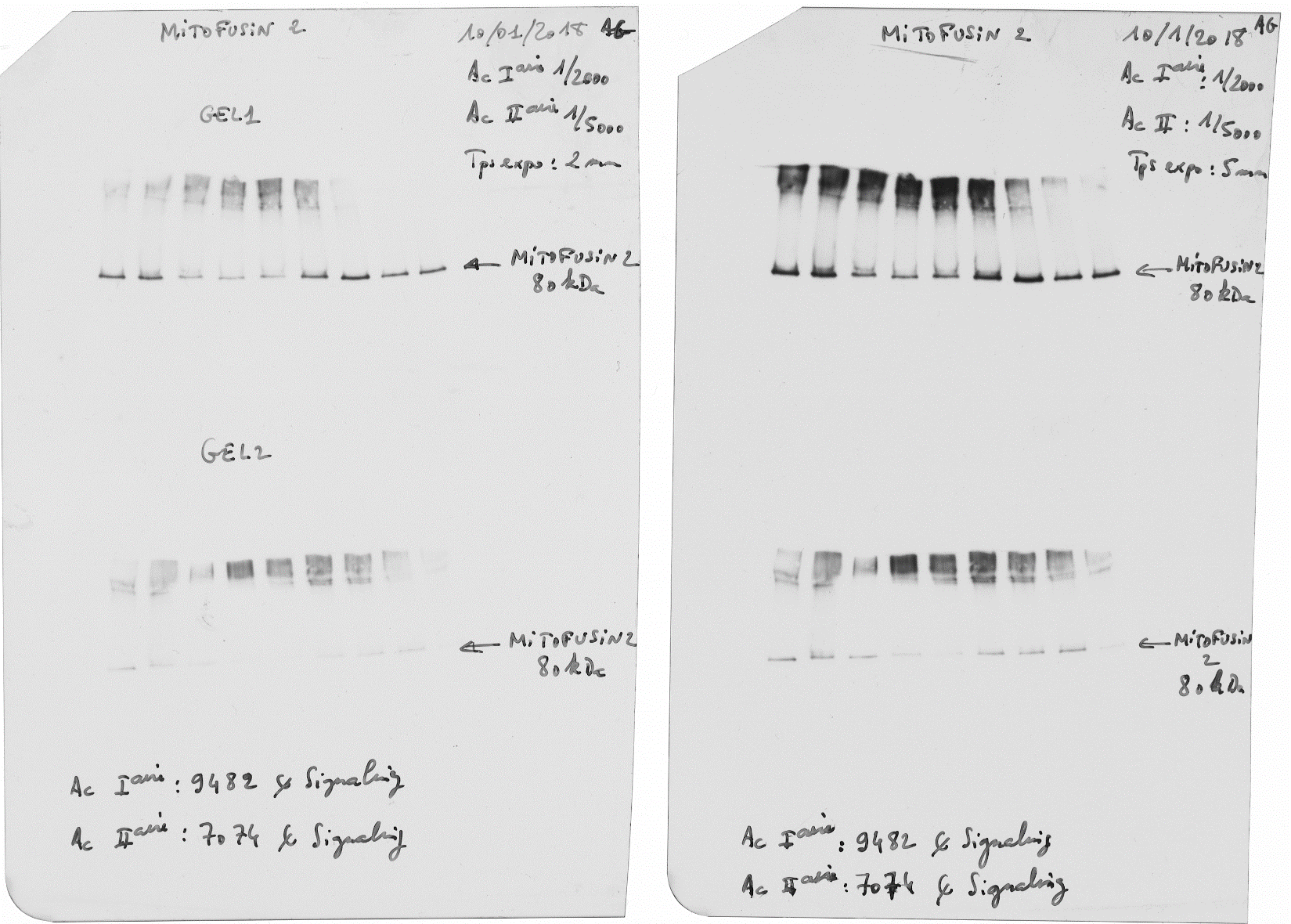

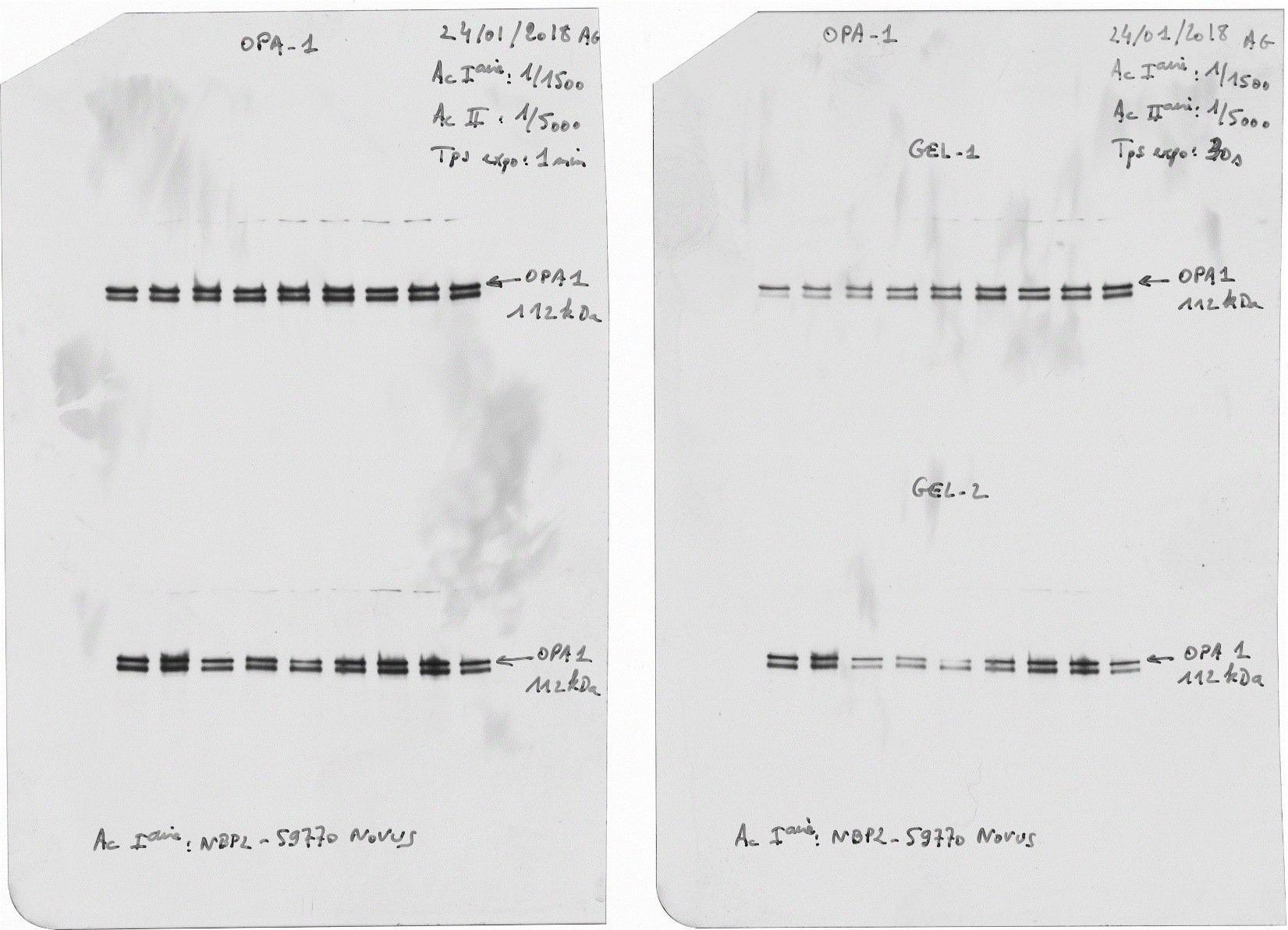


Mitofusin 1

Mitofusin 2

OPA 1

β-actin

**Control**

**LFA**

**ABA**

**Supplemental Fig. S5: Immunoblots for mitochondrial markers of fusion, mitofusin 1 and 2 and OPA1, in the hypothalamus.**

Representative immunoblots and densitometric analyses of mitofusin 1, mitofusin 2, OPA1 and β-actin at day 17 in the hypothalamus obtained from Control mice or mice with limitation of food access (LFA) or activity-based anorexia (ABA). (from 6 individuals /group).


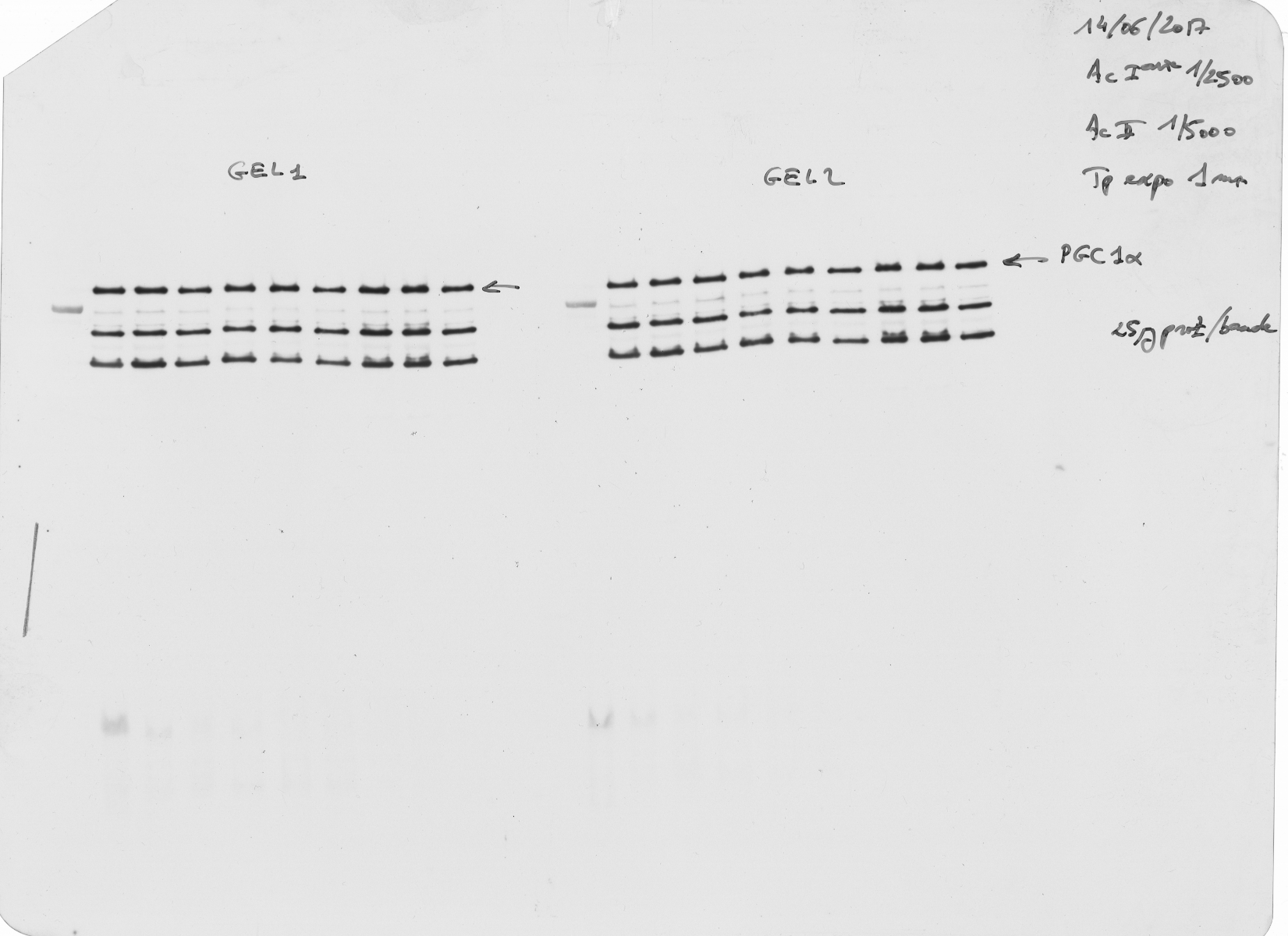

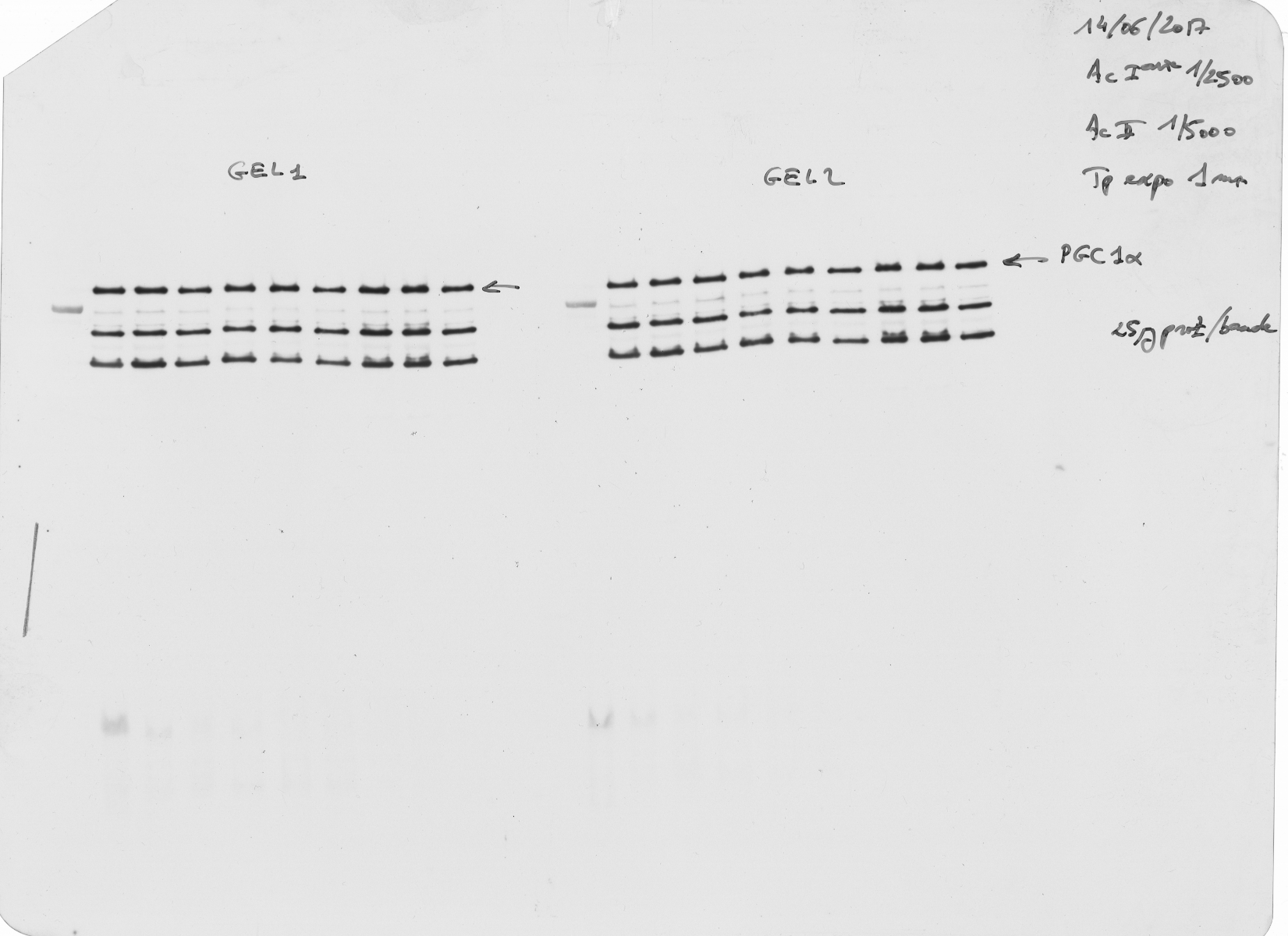

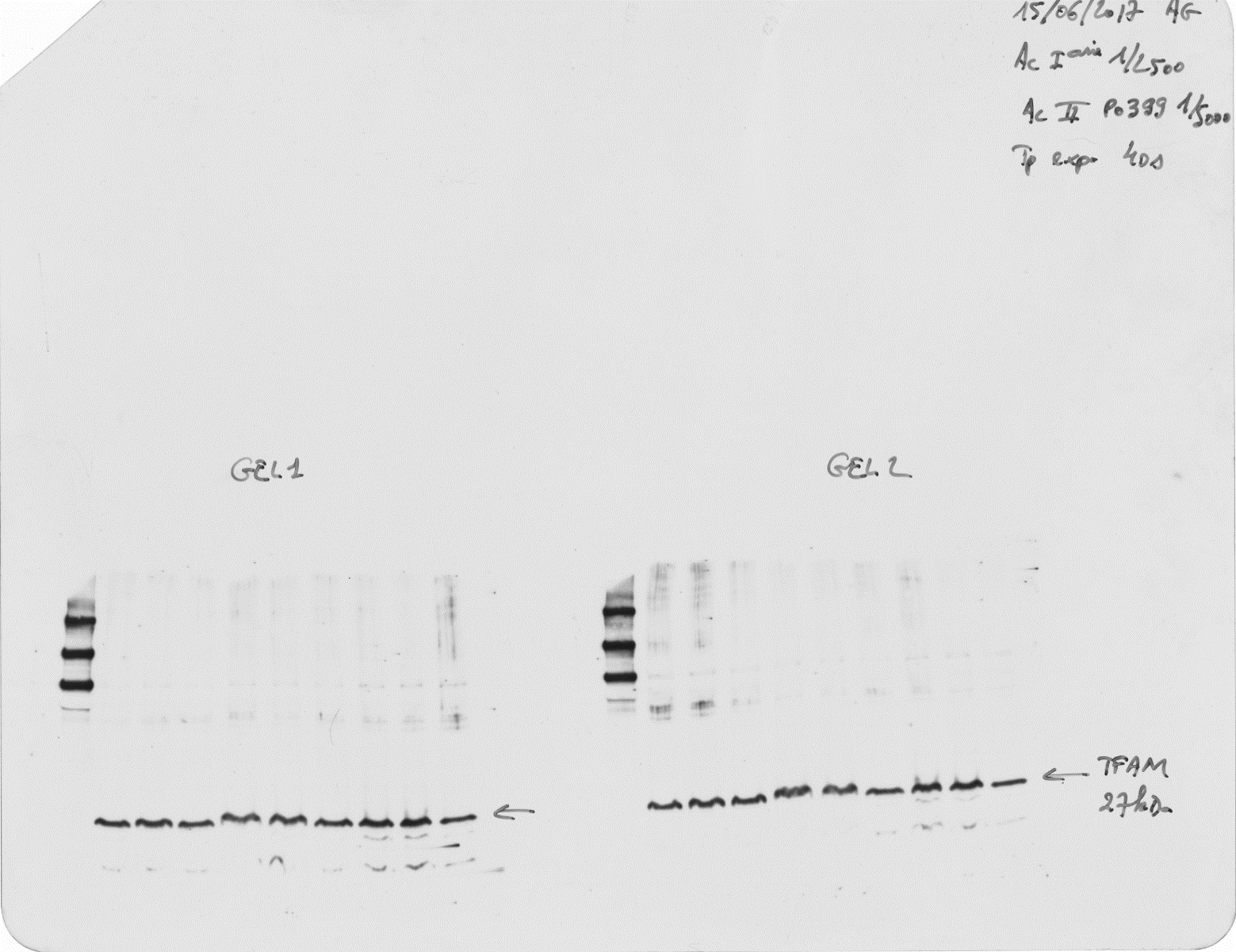

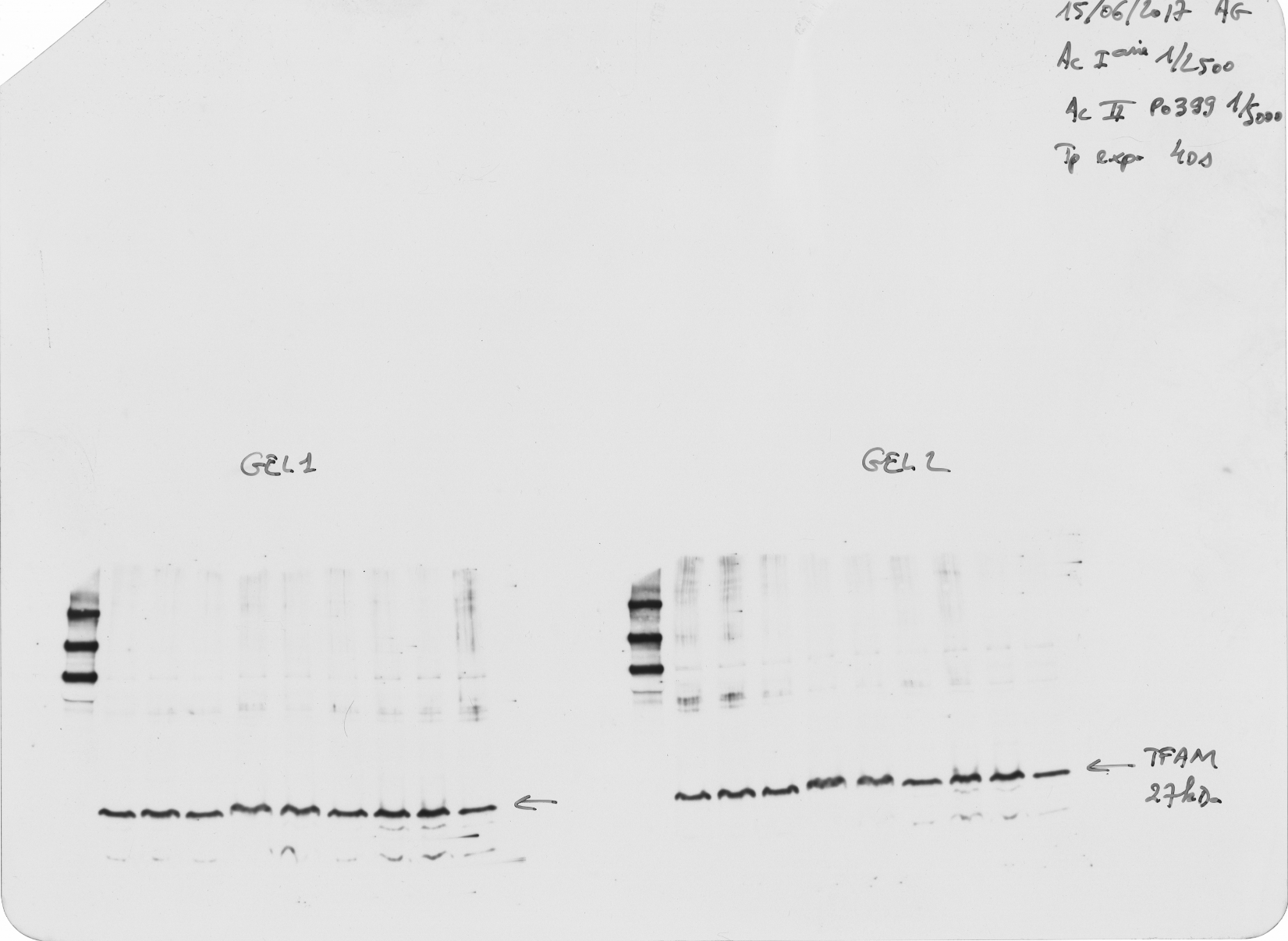

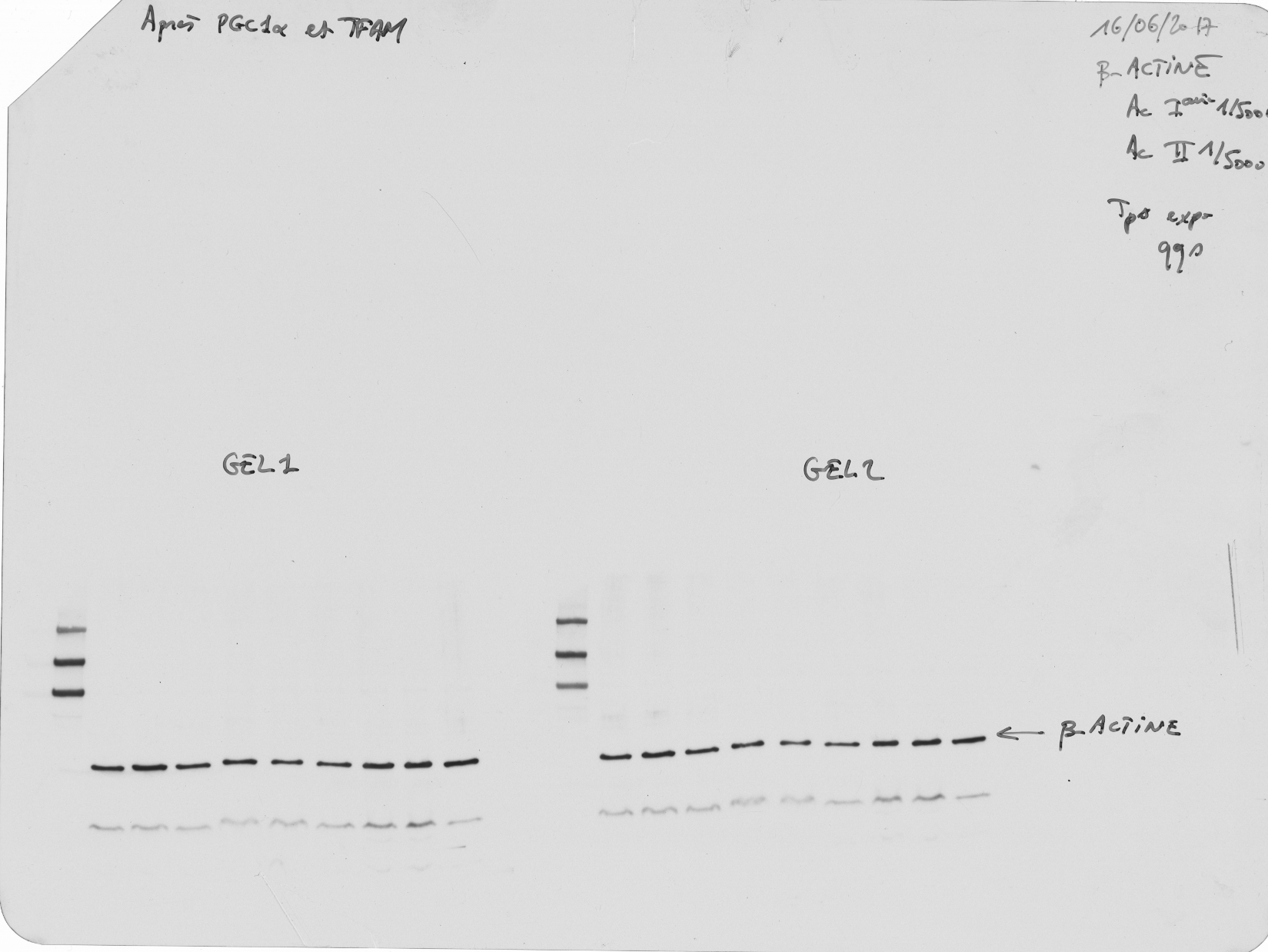

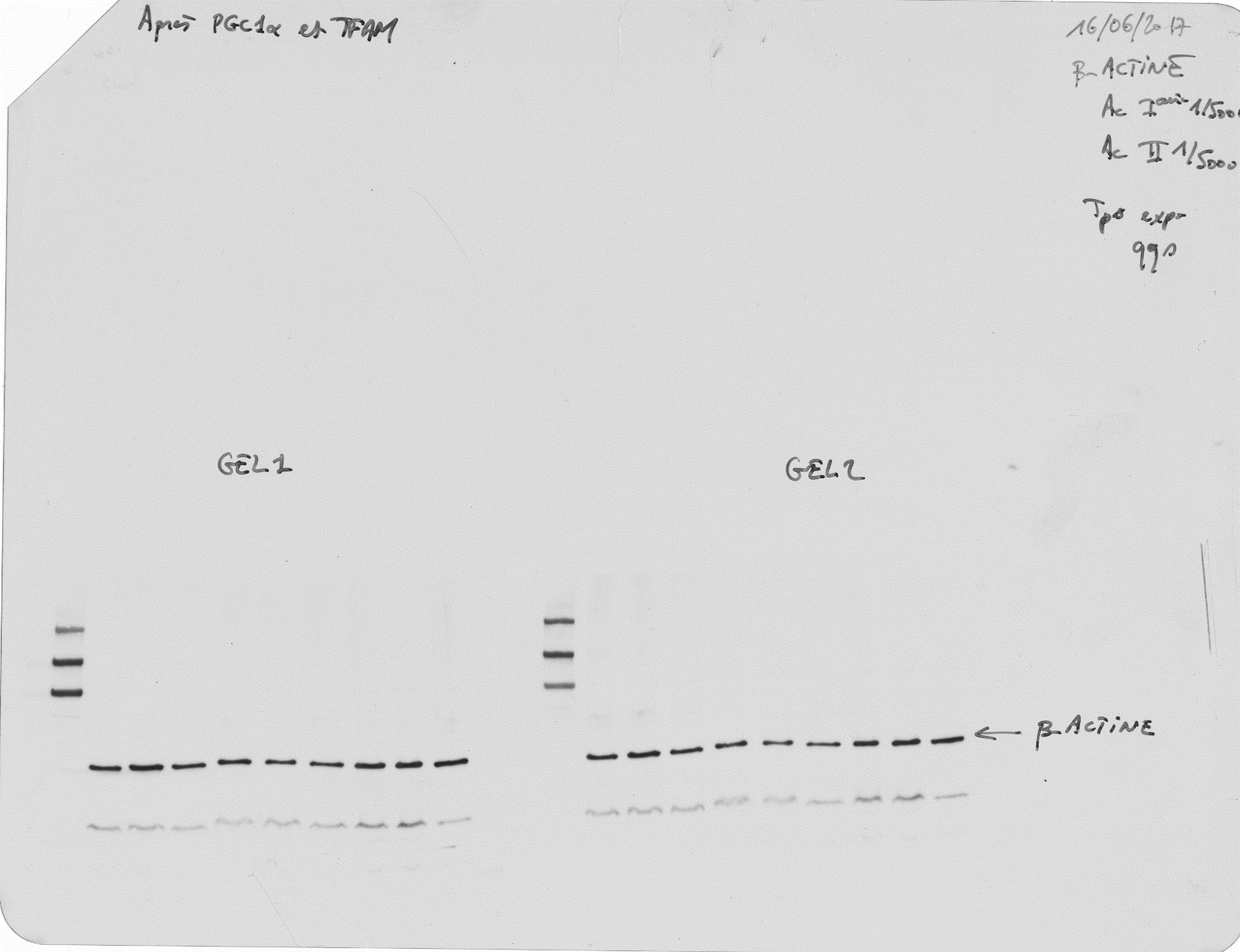


- PGC-1α

- TFAM

- β-actin

Control

LFA

ABA

Control

LFA

ABA

**Supplemental Fig. S6: Immunoblots for mitochondrial biogenesis markers, PGC-1α and TFAM, in the hypothalamus.**

Representative immunoblots for PGC-1^α^, TFAM and β-actin at day 17 in the hypothalamus obtained from Control mice or mice with limitation of food access (LFA) or activity-based anorexia (ABA). (from 6 individuals /group).


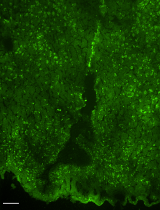

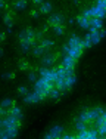


ABA


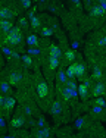

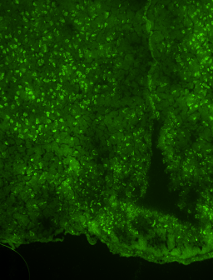

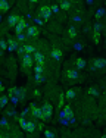

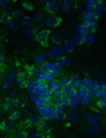


LFA


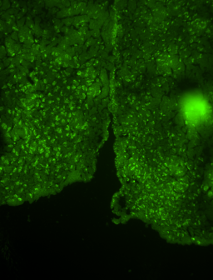

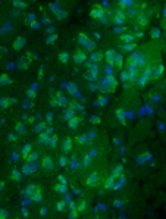

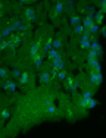


CT

125 µm

125 µm

125 µm

20 µm

20 µm

20 µm

20 µm

20 µm

20 µm

**Supplemental Fig. S7: Immunstaining of VDAC1 in the hypothalamus**

Representative microphotographs of VDAC1 expression (green) in the hypothalamus of Control mice (CT) or mice with limitation of food access (LFA) or activity-based anorexia (ABA) at day 17.


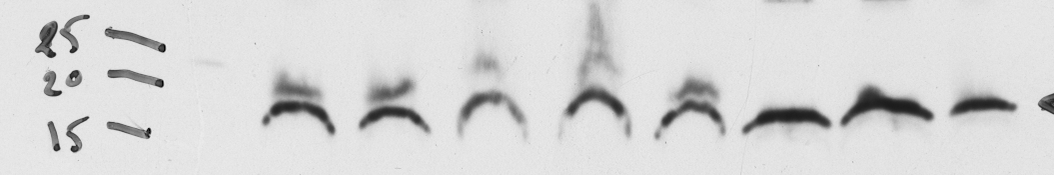


CT

LFA

ABA

**Supplemental Fig. S8: Gel used to show representative blots of LC3I and LC3II in figure 7**
